# Analytical Investigation of Non-Local Optoelectronic Photo-Thermoelastic Response in Fiber-Reinforced Anisotropic Silicon Using an Eigenvalue Framework

**DOI:** 10.3390/nano16171087

**Published:** 2026-08-31

**Authors:** Adel Emam, M. Yusuf, A. El-Dali, Zaki Mrzog Alaofi

**Affiliations:** 1 Faculty of Computer Studies, Arab Open University, Cairo 11211, Egypt; 2 Department of Mathematics, Faculty of Science, Capital University, Helwan, Cairo P.O. Box 11795, Egypt; mohammed.yousof@yahoo.com; 3 Department of Mathematics, Applied College at Mahail Aseer, King Khalid University, Abha 62521, Saudi Arabia; zaleawfi@kku.edu.sa

**Keywords:** non-local elasticity theory, optoelectronic photo-thermoelastic coupling, carrier transport dynamics, fiber-reinforced semiconductor media, eigenvalue–eigenvector method, anisotropic wave propagation, thermo-mechanical interactions, silicon half-space

## Abstract

This study aims to investigate the influence of non-local elasticity on the coupled optoelectronic photo-thermoelastic response of an anisotropic fiber-reinforced silicon half-space subjected to optical carrier excitation. A coupled analytical model is developed by incorporating non-local elasticity into a fiber-reinforced anisotropic semiconductor framework, where the thermal, carrier-density, displacement, and stress fields are fully coupled. After introducing the appropriate non-dimensional variables, the governing equations are transformed using the normal-mode technique into a system of ordinary differential equations and solved analytically through an eigenvalue-based vector–matrix approach. The novelty of the present work lies in examining the influence of the non-local parameter within a fiber-reinforced anisotropic semiconductor and performing a systematic comparison between fiber-reinforced and non-reinforced configurations under identical photothermal loading conditions. The numerical results demonstrate that increasing the non-local parameter produces pronounced changes in the mechanical response, including displacement amplitudes, stress distributions, and wave attenuation characteristics, whereas the temperature and carrier-density fields exhibit only slight variations within the investigated parameter range. Fiber reinforcement further influences the mechanical response by enhancing the structural stability and directional stiffness of the medium. The proposed analytical framework provides physical insight into the coupled effects of nonlocality and fiber reinforcement, with potential relevance to the analysis and design of semiconductor devices, optoelectronic and photonic structures, MEMS/NEMS, and smart fiber-reinforced composite materials operating under coupled thermo-mechanical and optical excitations.

## 1. Introduction

Recent advances in semiconductor technology and optoelectronic engineering have attracted considerable attention toward photo-thermoelastic interactions because of their important applications in laser-material processing, thermal sensing, microelectronic devices, and nano-scale engineering systems. Hobiny et al. [1] studied photo-thermoelastic waves in semiconductor materials under ramp-type heating, while Zenkour [2] developed generalized photo-thermoelastic formulations based on the three-phase-lag theory. Barone and Patterson [3] established simultaneous thermo- and photoelastic formulations for stress analysis, whereas Mondal et al. [4] investigated photo-thermoelastic wave propagation in the presence of memory responses. Abbas et al. [5] analyzed photo-thermal interactions in semiconductor materials with cylindrical cavities, while Awwad et al. [6] studied photo-thermoelastic behavior in functionally graded semiconductors subjected to laser pulse excitation. Moreover, Song et al. [7] discussed photothermal vibrations in semiconductor cantilevers near resonant frequencies, and Nasr and Abouelregal [8] investigated light absorption processes in generalized photo-thermoelastic semiconductor models. Gupta et al. [9] further examined vibrational analysis in thermo-piezo-photo-electric semiconductor media, whereas Alaofi and El-Dali [10] proposed a Multiphysics photo-thermo-hygroelastic model for semiconductor materials.

Fiber-reinforced and anisotropic materials have also attracted increasing interest because of their superior thermal and mechanical characteristics compared with conventional isotropic materials. Lu et al. [11] estimated the thermoelastic properties of polyimide fibers, while Akai et al. [12] evaluated thermoelastic temperature variations in carbon fiber-reinforced composites. Chaudhary et al. [13] investigated photothermal interactions in fiber-reinforced semiconducting media, whereas Saeed [14] studied photo-thermoelastic interactions under hyperbolic two-temperature theory. Escalante-Solís et al. [15] discussed the effect of fiber curvature on reinforced composites, while Alaofi et al. [16] analyzed coupled thermo-photoelastic fields in anisotropic fiber-reinforced silicon using an eigenvalue method. In addition, Abo-Dahab et al. [17] investigated wave reflections in fiber-reinforced thermoelastic media, whereas Kundu et al. [18] studied photothermal interactions in reinforced semiconducting structures subjected to moving thermal loads. Pitarresi et al. [19] and Quinlan et al. [20] also emphasized the importance of fiber reinforcement in improving thermoelastic responses and structural stability in composite materials. Recently, non-local thermoelasticity has emerged as an effective framework for describing thermal and elastic interactions at micro- and nano-scale dimensions where classical local theories become inadequate. Geetanjali et al. [21] investigated non-local photo-thermo-diffusive elastic media containing spherical cavities, while Ailawalia et al. [22] analyzed transitional stresses in non-local thermoelastic disks. Makkad et al. [23] studied non-local thermoviscoelastic bending behavior in nano-beams, whereas El-Dali and Othman [24] examined non-local effects in optoelectronic semiconductor materials. Alhassan et al. [25], Balwir et al. [26], and Sur [27] further investigated non-local thermoelastic interactions under different physical conditions. Moreover, Alshalhoub et al. [28], Sardar et al. [29], Banerjee et al. [30], and Kumar et al. [31] demonstrated the important influence of non-locality on the propagation and attenuation characteristics of coupled thermoelastic fields in semiconductor structures. Recent studies have further advanced the field of generalized thermoelasticity and semiconductor modeling by investigating different coupled thermo-mechanical phenomena, including photo-thermoelastic interactions, non-local effects, and anisotropic media [32,33,34,35,36]. These contributions provide valuable insights into the mathematical modeling and physical behavior of semiconductor materials and further motivate the development of the present non-local fiber-reinforced photo-thermoelastic formulation. Recent investigations have also extended these developments to advanced non-local and generalized thermoelastic models, emphasizing analytical modeling, wave propagation, heat transfer, and coupled multiphysical responses in semiconductor and engineering materials, thereby further enriching the current state of research in this field [37,38,39,40,41,42].

Despite the substantial progress achieved in the literature, the combined influence of non-local elasticity and anisotropic fiber reinforcement on coupled photo-thermoelastic semiconductor media has not yet been thoroughly investigated. A comprehensive analytical study addressing the effect of the non-local parameter in anisotropic fiber-reinforced semiconductor materials, together with a systematic comparison between reinforced and non-reinforced configurations, remains unavailable. Accordingly, the aim of the present work is to develop a non-local photo-thermoelastic model for an anisotropic fiber-reinforced semiconductor medium and to investigate the influence of the non-local parameter on the coupled thermoelastic response through a systematic comparison with the corresponding non-reinforced configuration. The governing equations are transformed into a vector–matrix differential system and solved analytically using the normal mode analysis technique. The novelty of the present study lies in (i) incorporating the non-local elasticity theory into an anisotropic fiber-reinforced semiconductor model, (ii) investigating the influence of the non-local parameter on the coupled thermoelastic response of the reinforced medium, and (iii) providing a detailed comparative analysis between fiber-reinforced and non-reinforced semiconductor configurations to quantify the role of fiber reinforcement under photo-thermal excitation. The obtained results provide useful physical insights into the design and optimization of advanced semiconductor structures, optoelectronic and photonic devices, micro- and nano-electromechanical systems (MEMS/NEMS), fiber-reinforced smart composite materials, and other engineering applications where coupled thermo-mechanical and photo-induced effects are significant.

## 2. Mathematical Formulation of the Non-Local Optoelectronic Photo-Thermoelastic Problem

**Figure 1:**
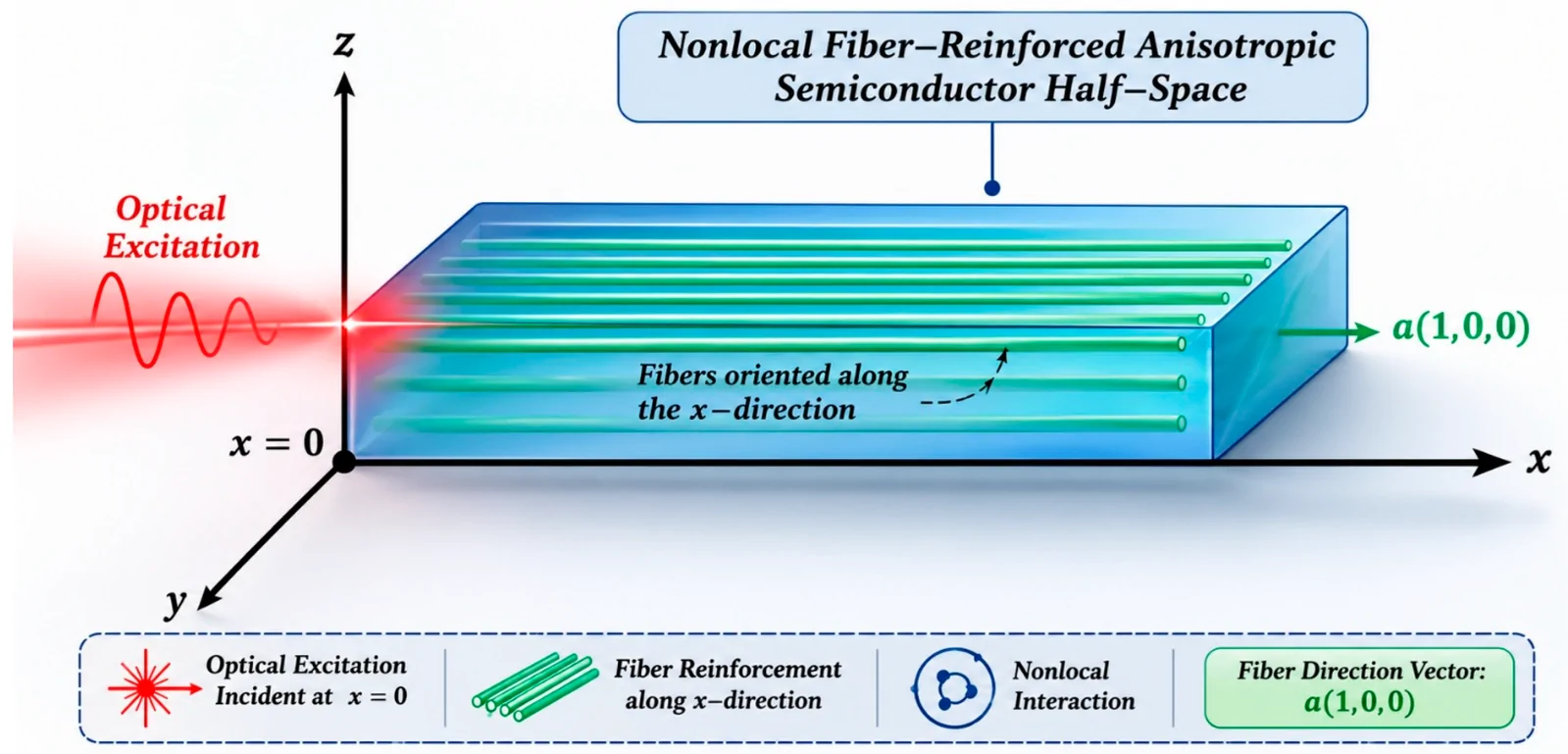
Schematic illustration of the non-local fiber-reinforced semiconductor half-space subjected to optoelectronic excitation, showing the coordinate system and fiber orientation.

In the present investigation, a two-dimensional non-local fiber-reinforced anisotropic semiconductor half-space is considered under optoelectronic excitation. The medium occupies the semi-infinite region x≥0, where the plane x=0 represents the boundary surface exposed to an incident optical source. A Cartesian coordinate system $\left(x,y,z\right)$ is adopted such that the x-axis extends normally into the medium, while the y-axis lies along the boundary surface. The reinforcing fibers are assumed to be uniformly distributed and aligned in the x-direction, introducing anisotropic characteristics into the semiconductor structure. The material is assumed to be homogeneous, linearly elastic, and isotropic in the absence of fiber reinforcement. The interaction between optical excitation and the semiconductor medium generates coupled thermal, elastic, and carrier-density fields due to the absorption of optical energy at the boundary surface. Consequently, excess carrier distributions and thermal disturbances propagate inside the medium, leading to coupled optoelectronic photo-thermoelastic interactions. Within the framework of the non-local generalized thermoelasticity theory and under the assumption of small deformations, the physical state of the medium is characterized by the temperature distribution T(x,y,t), carrier density N(x,y,t), and displacement components u(x,y,t) and v(x,y,t). The governing equations are formulated to describe the coupled behavior of thermal waves, elastic deformations, and carrier transport processes in the reinforced semiconductor half-space. Figure 1 illustrates the geometrical configuration of the problem, including the coordinate system, the direction of optical excitation, and the alignment of the reinforcing fibers embedded inside the non-local semiconductor medium.

To characterize the coupled mechanical and photo-thermoelastic behavior of the considered semiconductor medium, the constitutive equations of a fiber-reinforced anisotropic thermoelastic material are introduced. These relations describe the interaction between elastic deformation, thermal variations, and excess carrier concentration generated by optical excitation. Following the generalized formulations reported in [11,15,19,20], the non-local constitutive relation is expressed as

(1) $$(1-\xi^{2}\nabla^{2})\sigma_{ij}=C_{ijkl}e_{kl}-\beta_{ij}T-\eta_{ij}N.$$

where $\sigma_{ij}$ denotes the stress tensor components, $C_{ijkl}$ is the elastic stiffness tensor, and $e_{kl}$ represents the infinitesimal strain tensor. The parameters $\beta_{ij}$ and $\eta_{ij}$ correspond to thermoelastic and carrier-elastic coupling coefficients, respectively, while ξ denotes the non-local parameter. In the presence of fiber reinforcement, the constitutive equations become direction-dependent due to the preferred orientation of reinforcing fibers embedded within the semiconductor matrix. Accordingly, the constitutive relation may be written in the following form [11,15,17]

(2) $$\sigma_{ij}=\lambda e_{kk}\delta_{ij}+2\mu_{T}e_{ij}+\alpha \left(a_{k}a_{m}e_{km}\delta_{ij}+a_{i}a_{j}e_{kk}\right)+\beta a_{k}a_{m}e_{km}a_{i}a_{j}+2(\mu_{L}-\;\mu_{T})(a_{i}a_{k}e_{kj}+a_{j}a_{k}e_{ki})-\beta_{ij}T-\eta_{ij}N.$$

The constitutive relation adopted in Equation (2) follows the generalized fiber-reinforced continuum framework originally proposed by Spencer and subsequently employed in thermoelastic analyses of reinforced composite media. Within this formulation, the parameters α and β quantify the additional stiffness contributions arising from the presence and orientation of the reinforcing fibers, whereas $\mu_{L}$ and $\mu_{T}$ denote the effective longitudinal and transverse shear moduli, respectively, reflecting the anisotropic mechanical response of the reinforced medium. These parameters enable the constitutive model to capture the directional dependence of stress transfer and deformation induced by fiber reinforcement while remaining fully consistent with the generalized thermoelastic formulation adopted in the present work. Therefore, the stress components reduce to the following expressions [15,20]

(3) $$(1-\xi^{2}\nabla^{2})\sigma_{xx}=A_{11}\frac{\partial u}{\partial x}+A_{12}\frac{\partial v}{\partial y}-\beta_{11}T-\eta_{11}N$$

(4) $$(1-\xi^{2}\nabla^{2})\sigma_{yy}=A_{12}\frac{\partial u}{\partial x}+A_{13}\frac{\partial v}{\partial y}-\beta_{22}T-\eta_{22}N$$

(5) $$(1-\xi^{2}\nabla^{2})\sigma_{xy}=A_{14}\left(\frac{\partial u}{\partial y}+\frac{\partial v}{\partial x}\right).$$

where u and v denote the displacement components in the x- and y-directions, respectively, and $A_{ij}$ are the effective elastic coefficients of the reinforced anisotropic medium. The effective thermoelastic and carrier-elastic coupling coefficients are defined by

$$\beta_{11}=\left(2\lambda +4\mu_{L}-2\mu_{T}+3\alpha +\beta \right)\alpha_{11}+\left(\lambda +\alpha \right)\alpha_{22},$$

$$\beta_{22}=(2\lambda +\alpha )\alpha_{11}+(\lambda +2\mu_{T})\alpha_{22},$$

$$\eta_{11}=\left(2\lambda +4\mu_{L}-2\mu_{T}+3\alpha +\beta \right)\xi_{11}+\left(\lambda +\alpha \right)\xi_{22},$$

$$\eta_{22}=(2\lambda +\alpha )\xi_{11}+(\lambda +2\mu_{T})\xi_{22}.$$

where $\alpha_{ij}$ denote the thermal expansion coefficients, while $\xi_{ij}$ represent the carrier-expansion parameters. Finally, the effective elastic parameters associated with the reinforced semiconductor medium are given by [11,17]

$$A_{11}=\lambda +2\mu_{T}+2\alpha +\beta +4(\mu_{L}-\mu_{T}),\;A_{12}=\lambda +\alpha ,\;A_{13}=\lambda +2\mu_{T},\;A_{14}=\mu_{L}.$$

The mechanical behavior of the non-local fiber-reinforced semiconductor medium is governed by the generalized equations of motion, which describe the dynamic coupling between the stress field and the displacement components. Following the formulations adopted in Refs. [15,17,20,22], the equations of motion in the absence of body moments can be written as

(6) $$\rho u_{tt}=\left(\frac{\partial \sigma_{xx}}{\partial x}+\frac{\partial \sigma_{xy}}{\partial y}\right)$$

(7) $$\rho v_{tt}=\left(\frac{\partial \sigma_{xy}}{\partial x}+\frac{\partial \sigma_{yy}}{\partial y}\right).$$

where ρ denotes the mass density of the medium, while u and v represent the displacement components along the x- and y-directions, respectively. Applying the non-local operator to the governing equations yields

(8) $$(1-\xi^{2}\nabla^{2})\rho u_{tt}=\left(\frac{\partial (1-\xi^{2}\nabla^{2})\sigma_{xx}}{\partial x}+\frac{\partial (1-\xi^{2}\nabla^{2})\sigma_{xy}}{\partial y}\right)$$

(9) $$(1-\xi^{2}\nabla^{2})\rho v_{tt}=\left(\frac{\partial (1-\xi^{2}\nabla^{2})\sigma_{xy}}{\partial x}+\frac{\partial (1-\xi^{2}\nabla^{2})\sigma_{yy}}{\partial y}\right).$$

Substituting the constitutive relations given in Equations (3)–(5) into the above equations, the coupled equations of motion for the reinforced anisotropic semiconductor half-space are obtained as [15,20]

(10) $$(1-\xi^{2}\nabla^{2})\rho u_{tt}=A_{11}u_{xx}+A_{14}u_{yy}+(A_{12}+A_{14})v_{xy}-\beta_{11}T_{x}-\eta_{11}N_{x}$$

(11) $$(1-\xi^{2}\nabla^{2})\rho v_{tt}=(A_{12}+A_{14})u_{xy}+A_{14}v_{xx}+A_{13}v_{yy}-\beta_{22}T_{y}-\eta_{22}N_{y}$$

where the subscripts attached to the field variables denote partial differentiation with respect to the corresponding spatial or temporal coordinates. These equations describe the coupled propagation of elastic disturbances, thermal waves, and carrier-density variations inside the non-local fiber-reinforced semiconductor medium under optoelectronic excitation. The presence of the non-local parameter modifies the inertial and stress-transfer mechanisms, whereas the reinforcing fibers introduce anisotropic stiffness effects that significantly influence the wave propagation characteristics of the medium. The interaction between thermal, elastic, and carrier-density fields generates additional coupling effects that strongly influence the heat-transfer mechanism inside the semiconductor medium. Within the framework of generalized photo-thermoelastic, the heat conduction equation for a non-local fiber-reinforced anisotropic semiconductor medium can be formulated as [15,22]

(12) $$K_{ij}T_{,ij}+\frac{E_{g}}{\tau }N=\rho C_{E}T_{t}+T_{0}\beta_{ij}u_{i,jt}$$

where $K_{ij}$ denotes the anisotropic thermal conductivity tensor associated with the reinforced medium. The parameter $E_{g}$ represents the semiconductor energy-gap constant responsible for carrier generation effects, while τ is the carrier lifetime parameter related to the recombination process. Moreover, $\rho C_{E}$ corresponds to the volumetric heat capacity of the material, where $C_{E}$ is the specific heat at constant strain. The quantity $T_{0}$ denotes the reference temperature in the natural equilibrium state. The last term in Equation (12) represents the thermoelastic coupling contribution between the temperature field and the strain-rate field through the coefficients $\beta_{ij}$. This coupling becomes more pronounced in reinforced anisotropic media due to the directional dependence introduced by the embedded fibers.

For the present two-dimensional configuration, Equation (12) reduces to the following form:

(13) $$K_{11}T_{xx}+K_{22}T_{yy}+\frac{E_{g}}{\tau }N=\rho C_{E}T_{t}+T_{0}\left(\beta_{11}u_{x,t}+\beta_{22}v_{y,t}\right)$$

where $K_{11}$ and $K_{22}$ are the effective thermal conductivities along the principal material directions. Equation (13) indicates that the temperature distribution inside the semiconductor medium is affected not only by anisotropic heat conduction, but also by carrier-generation effects and the thermoelastic coupling associated with the deformation rates of the reinforced structure. In addition, the presence of the reinforcing fibers modifies the directional heat-transfer characteristics, leading to anisotropic thermal-wave propagation within the semiconductor half-space. Under optoelectronic excitation, the semiconductor medium absorbs incident optical energy, leading to the generation of excess charge carriers inside the material. These carriers undergo diffusion and recombination processes, which are strongly coupled with both the thermal and elastic fields. Consequently, the carrier-density distribution plays a significant role in determining the overall photo-thermoelastic response of the reinforced semiconductor medium. Based on the generalized carrier transport theory in semiconductors [1,5,25], the evolution equation governing the excess carrier density can be expressed as

(14) $$\frac{\partial N}{\partial t}=D_{E}\nabla^{2}N-\frac{N}{\tau }+\kappa T$$

where $D_{E}$ denotes the carrier diffusion coefficient responsible for the spatial transport of carriers inside the semiconductor medium. The term $\frac{N}{\tau }$ represents the carrier recombination effect, with τ being the carrier lifetime parameter. Moreover, κ is the thermo-carrier coupling coefficient associated with the generation of carriers due to temperature variations. These equations indicate that the carrier transport process is significantly influenced by the optoelectronic excitation mechanism and its interaction with the thermal disturbances inside the anisotropic fiber-reinforced semiconductor half-space.

## 3. Non-Dimensional Formulation of the Coupled Non-Local Photo-Thermoelastic System

To simplify the mathematical structure of the governing equations and facilitate the analytical treatment of the coupled problem, a suitable non-dimensionalization procedure is introduced. This transformation reduces the number of material parameters and converts the governing equations into compact normalized forms suitable for investigating the interactions among elastic deformation, thermal transport, and carrier-density propagation in the reinforced semiconductor medium. Similar normalization procedures were adopted in [2,15,20,22]. Accordingly, the following non-dimensional variables are introduced

(15) $$\left(x^{\prime },y^{\prime },u^{\prime },v^{\prime },\xi^{\prime }\right)=\frac{\left(x,y,u,v,\xi \right)}{C_{T}t^{*}},t^{\prime }=\frac{t}{t^{*}},{(T}^{\prime },N^{\prime })=\frac{{(\beta }_{xx},\;\;{\;\eta }_{xx})\;}{A_{11}}\;T,\sigma^{\prime }=\frac{\sigma }{A_{11}},C_{T}^{2}=\frac{A_{11}}{\rho },\;t^{*}=\frac{K_{11}}{\rho C_{E}C_{T}^{2}}.$$

Here, $C_{T}=\sqrt{A_{11}/\rho }$ denotes the characteristic elastic-wave velocity obtained directly from the equation of motion, representing the natural propagation speed of elastic disturbances in the fiber-reinforced semiconductor medium. This characteristic velocity is therefore adopted as the reference velocity in the non-dimensionalization procedure. Accordingly, the characteristic time scale is defined as $t^{*}=K_{11}/\left(\rho C_{E}C_{T}^{2}\right)$, which reflects the characteristic thermal diffusion time associated with the coupled photo-thermoelastic process and provides a physically consistent normalization of the coupled governing equations. After introducing the above dimensionless variables, the prime notation is omitted hereafter for simplicity, and the normalized governing equations are obtained in the following form. Following the non-dimensionalization procedure described in Refs. [15,20,22], the governing equations reduce to

(16) $$\frac{\partial^{2}u}{\partial x^{2}}=-a_{5}\frac{\partial^{2}u}{\partial y^{2}}+\left(1-\xi^{2}\left(\frac{\partial^{2}}{\partial x^{2}}+\frac{\partial^{2}}{\partial y^{2}}\right)\right)\frac{\partial^{2}u}{\partial t^{2}}-a_{6}\frac{\partial^{2}v}{\partial x\partial y}+\frac{\partial T}{\partial x}+\frac{\partial N}{\partial x}$$

(17) $$\frac{\partial^{2}v}{\partial x^{2}}=-a_{7}\frac{\partial^{2}v}{\partial y^{2}}+a_{8}\left(1-\xi^{2}\left(\frac{\partial^{2}}{\partial x^{2}}+\frac{\partial^{2}}{\partial y^{2}}\right)\right)\frac{\partial^{2}v}{\partial t^{2}}-a_{9}\frac{\partial^{2}u}{\partial x\partial y}+a_{10}\frac{\partial T}{\partial y}+a_{11}\frac{\partial N}{\partial y}.$$

(18) $$\frac{\partial^{2}T}{\partial x^{2}}=-a_{12}\frac{\partial^{2}T}{\partial y^{2}}+\frac{\partial T}{\partial t}-a_{13}N+a_{14}\frac{\partial }{\partial t}\left(\frac{\partial u}{\partial x}\right)+a_{15}\frac{\partial }{\partial t}\left(\frac{\partial v}{\partial y}\right).$$

(19) $$\frac{\partial^{2}N}{\partial x^{2}}=-\frac{\partial^{2}N}{\partial y^{2}}+a_{16}\frac{\partial N}{\partial t}+a_{17}N-a_{18}T$$

The normalized stress components can therefore be written as

(20) $$\left(1-\xi^{2}\nabla^{2}\right)\sigma_{xx}=\frac{\partial u}{\partial x}+a_{1}\frac{\partial v}{\partial y}-(T+N)$$

(21) $$\left(1-\xi^{2}\nabla^{2}\right)\sigma_{yy}=a_{1}\frac{\partial u}{\partial x}+a_{2}\frac{\partial v}{\partial y}-a_{3}T-a_{4}N$$

(22) $$\left(1-\xi^{2}\nabla^{2}\right)\sigma_{xy}=a_{5}\left(\frac{\partial u}{\partial y}+\frac{\partial v}{\partial x}\right)$$

The dimensionless coefficients $a_{i}$ introduced in the above formulation represent compact combinations of the physical and material parameters governing the coupled non-local photo-thermoelastic behavior of the reinforced semiconductor medium. These parameters simplify the mathematical representation of the system and provide clear physical interpretations for the interactions between the elastic, thermal, and carrier-density fields. The parameter $a_{1}=\frac{A_{12}}{A_{11}}$ represents the normalized coupling between the longitudinal and transverse deformation components, whereas $a_{2}=\frac{A_{13}}{A_{11}}$ characterizes the contribution of transverse normal deformation relative to the principal elastic stiffness. The coefficient $a_{3}=\frac{\beta_{yy}}{\beta_{xx}}$ describes the anisotropic thermoelastic coupling effect associated with directional thermal expansion, while $a_{4}=\frac{\eta_{yy}}{\eta_{xx}}$ measures the influence of carrier-density coupling along different material directions. Furthermore, $a_{5}=\frac{A_{14}}{A_{11}}$ denotes the normalized shear stiffness parameter governing the shear deformation contribution within the reinforced medium.

The parameters $\gamma_{1}=\frac{\rho }{A_{11}t^{*2}}$ and $\gamma_{2}=\frac{\rho }{A_{14}t^{*2}}$ correspond to the normalized inertial coefficients associated with longitudinal and shear elastic responses, respectively. In addition, the parameter $a_{6}=\frac{A_{12}+A_{14}}{A_{11}}$ characterizes the coupling between normal and shear deformation components appearing in the governing displacement equations, whereas $a_{7}=\frac{A_{13}}{A_{14}}$ represents the ratio between transverse and shear stiffness contributions. The coefficient $a_{8}=\frac{C_{T}^{2}\rho }{A_{14}}$ defines the normalized inertial parameter related to wave propagation effects in the reinforced semiconductor structure. Moreover, $a_{9}=\frac{A_{12}+A_{14}}{A_{14}}$ quantifies the interaction between displacement gradients in different spatial directions. The parameters $a_{10}=\frac{A_{11}\beta_{yy}}{A_{14}\beta_{xx}}$ and $a_{11}=\frac{A_{11}\eta_{yy}}{A_{14}\eta_{xx}}$ represent the relative contributions of thermal and carrier-density coupling effects to the transverse deformation field, respectively. The coefficient $a_{12}=\frac{K_{22}}{K_{11}}$ describes the anisotropy in thermal conductivity along the principal material directions. The parameter $a_{13}=\frac{t^{*2}E_{g}\beta_{xx}}{K_{11}\eta_{xx}}$ characterizes the influence of carrier recombination and energy-gap effects on the thermal field, whereas $a_{14}=\frac{\beta_{xx}^{2}C_{T}T_{0}t^{*}}{A_{11}K_{11}}$ measures the coupling between temperature variations and time-dependent elastic deformation. Similarly, the coefficient $a_{15}=\frac{\beta_{xx}\beta_{yy}C_{T}T_{0}t^{*}}{A_{11}K_{11}}$ accounts for the combined thermoelastic coupling contribution in both spatial directions. Finally, the parameter $a_{16}=\frac{C_{T}^{2}t^{*}}{D_{E}}$ represents the normalized carrier-diffusion parameter controlling the transport rate of excess carriers, while $a_{17}=\frac{C_{T}^{2}t^{*2}}{D_{E}\tau }$ characterizes the relative influence of carrier recombination processes. The coefficient $a_{18}=\frac{C_{T}^{2}\eta_{xx}\kappa t^{*2}}{D_{E}\beta_{xx}}$ describes the coupling between thermal disturbances and carrier-generation mechanisms induced by optoelectronic excitation.

## 4. Analytical Solution Using the Normal Mode Technique

Although the present problem is transient, the normal mode technique provides a rigorous analytical framework for representing the temporal and spatial dependence of the coupled photo-thermoelastic fields. By introducing a harmonic dependence with respect to time and the transverse spatial coordinate, the governing partial differential equations are transformed into an equivalent system of ordinary differential equations with respect to the spatial coordinate x, while the transient characteristics remain fully represented through the complex frequency parameter ω. This approach has been widely adopted in generalized thermoelasticity and semiconductor models because of its mathematical efficiency, analytical tractability, and physical consistency. Accordingly, the temperature, carrier density, displacement, and stress fields are expressed in the following exponential normal-mode form [5,15,28]:

(23) $$(T,N,u,v,\sigma )(x,y,t)=(T^{*},N^{*},u^{*},v^{*},\sigma^{*})(x)\;e^{\omega t+iay}$$

where a denotes the wavenumber associated with the spatial variation along the y-direction, while ω represents the complex frequency parameter governing the temporal evolution of the coupled fields. The real part of ω characterizes the propagation and oscillatory behavior of the waves, whereas the imaginary part is associated with attenuation and stability characteristics of the physical system. Consequently, negative values of the real part of ω ensure the boundedness and stability of the obtained solution as time progresses. Furthermore, $i=\sqrt{-1}$ denotes the imaginary unit. The quantities $T^{*}$, $N^{*}$, $u^{*}$, $v^{*}$, and $\sigma^{*}$ represent the amplitudes of the corresponding physical fields and depend only on the spatial coordinate x. Substituting the normal mode expressions given in Equation (23) into the previously derived non-dimensional governing Equations (16)–(22) transforms the coupled system into a set of ordinary differential equations in the transformed domain. Following the transformation procedure described in Refs. [20,22,28], the thermal and carrier-density equations reduce to

(24) $$\frac{d^{2}T^{*}}{dx^{2}}=E_{7}T^{*}+a_{13}N^{*}+E_{8}\frac{du^{*}}{dx}+E_{9}v^{*}$$

(25) $$\frac{d^{2}N^{*}}{dx^{2}}=E_{10}N^{*}-a_{18}T^{*}$$

While the transformed displacement equations take the form

(26) $$\frac{d^{2}u^{*}}{dx^{2}}=\frac{E_{1}}{P_{1}}u^{*}-\frac{E_{2}}{P_{1}}\frac{dv^{*}}{dx}+\frac{E_{3}}{P_{1}}\left(\frac{dT^{*}}{dx}+\frac{dN^{*}}{dx}\right)$$

(27) $$\frac{d^{2}v^{*}}{dx^{2}}=\frac{E_{3}}{P_{2}}v^{*}-\frac{E_{4}}{P_{2}}\frac{du^{*}}{dx}+\frac{E_{5}}{P_{2}}T^{*}+\frac{E_{6}}{P_{2}}N^{*}$$

The transformed stress components are therefore obtained as

(28) $$\xi^{2}\frac{d^{2}\sigma_{xx}^{*}}{dx^{2}}=\left(1+a^{2}\xi^{2}\right)\sigma_{xx}^{*}-Du^{*}-iaa_{1}v^{*}+T^{*}+N^{*}$$

(29) $$\xi^{2}\frac{d^{2}\sigma_{yy}^{*}}{dx^{2}}=\left(1+a^{2}\xi^{2}\right)\sigma_{yy}^{*}-a_{1}Du^{*}-iaa_{2}v^{*}+a_{3}T^{*}+a_{4}N^{*}$$

(30) $$\xi^{2}\frac{d^{2}\sigma_{xy}^{*}}{dx^{2}}=\left(1+a^{2}\xi^{2}\right)\sigma_{xy}^{*}-iaa_{5}u^{*}-a_{5}Dv^{*}.$$

where $D=\frac{d}{dx}$, denotes the differential operator with respect to the spatial coordinate x. The coefficients $E_{i}$ appearing in the transformed equations represent compact combinations of the normalized material and physical parameters governing the coupled non-local photo-thermoelastic interactions. These coefficients are defined as

$$\begin{array}{c} \;E_{1}=\omega^{2}+a^{2}\left(a_{5}+\xi^{2}\omega^{2}\right),\;E_{2}=iaa_{6},\;E_{3}=\left(a^{2}a_{7}+a_{8}\omega^{2}+a^{2}a_{8}\xi^{2}\omega^{2}\right),\;E_{4}=iaa_{9},E_{5}=iaa_{10},\;E_{6}=iaa_{11},\;E_{7}=\\ a^{2}a_{12}+\omega ,\;E_{8}=a_{14}\;\omega ,\;E_{9}=iaa_{15},\;E_{10}=a^{2}+a_{17}+a_{16}\omega ,\;P_{1}=1+\xi^{2}\omega^{2},\;P_{2}=1+a_{8}\xi^{2}\omega^{2}. \end{array}$$

These transformed equations provide a compact mathematical framework suitable for constructing the characteristic equation and deriving the complete analytical solution of the coupled non-local photo-thermoelastic problem. The obtained formulation clearly demonstrates the influence of nonlocality, anisotropic fiber reinforcement, thermal coupling, and carrier transport mechanisms on the propagation characteristics and attenuation behavior of waves inside the optoelectronically excited semiconductor half-space.

## 5. State-Space Matrix Formulation and Spectral Eigenvalue Analysis

Following the application of the normal mode transformation, the governing equations of the coupled non-local photo-thermoelastic system Equations (16)–(22) are reduced to a set of ordinary differential equations with respect to the spatial coordinate x, Equations (24)–(27). To construct a systematic analytical solution, the transformed system is rewritten in the form of a first-order state-space matrix differential equation. Such a formulation provides a convenient mathematical framework for investigating the propagation characteristics, attenuation behavior, and stability of coupled thermoelastic waves in the anisotropic fiber-reinforced semiconductor half-space. Similar matrix differential approaches have been employed in generalized thermoelastic and semiconductor analyses [2,15,20]. To transform the governing equations into a first-order differential system, the following auxiliary state variables are introduced:

(31) $$V_{1}=T^{*},V_{2}=N^{*},V_{3}=u^{*},V_{4}=v^{*},\;V_{5}=\frac{dT^{*}}{dx},V_{6}=\frac{dN^{*}}{dx},V_{7}=\frac{du^{*}}{dx},V_{8}=\frac{dv^{*}}{dx}.$$

Using these definitions together with Equations (24)–(27), the transformed governing equations can be rewritten as the following coupled first-order system

(32) $$\frac{dV_{1}}{dx}=V_{5},\frac{dV_{2}}{dx}=V_{6},\frac{dV_{3}}{dx}=V_{7},\frac{dV_{4}}{dx}=V_{8}$$

(33) $$\frac{dV_{5}}{dx}=E_{7}V_{1}+a_{13}V_{2}+E_{8}V_{7}+E_{9}V_{4}$$

(34) $$\frac{dV_{6}}{dx}=E_{10}V_{2}-a_{18}V_{1}$$

(35) $$\frac{dV_{7}}{dx}=\frac{E_{1}}{P_{1}}V_{3}-\frac{E_{2}}{P_{1}}V_{8}+\frac{1}{P_{1}}\left(V_{5}+V_{6}\right)$$

(36) $$\frac{dV_{8}}{dx}=\frac{E_{3}}{P_{2}}V_{4}-\frac{E_{4}}{P_{2}}V_{7}+\frac{E_{5}}{P_{2}}V_{1}+\frac{E_{6}}{P_{2}}V_{2}$$

The above system may therefore be expressed in the compact state-space matrix form

(37) $$\frac{dV}{dx}=AV$$

Where the state vector is defined by

(38) $$V=(V_{1},V_{2},V_{3},V_{4},V_{5},V_{6},V_{7},V_{8}{)}^{T}$$

And the corresponding coefficient matrix A takes the form

(39) $$A=\left(\begin{array}{cccccccc} 0 & 0 & 0 & 0 & 1 & 0 & 0 & 0\\ 0 & 0 & 0 & 0 & 0 & 1 & 0 & 0\\ 0 & 0 & 0 & 0 & 0 & 0 & 1 & 0\\ 0 & 0 & 0 & 0 & 0 & 0 & 0 & 1\\ E_{7} & a_{13} & 0 & E_{9} & 0 & 0 & E_{8} & 0\\ -a_{18} & E_{10} & 0 & 0 & 0 & 0 & 0 & 0\\ 0 & 0 & \frac{E_{1}}{P_{1}} & 0 & \frac{1}{P_{1}} & \frac{1}{P_{1}} & 0 & -\frac{E_{2}}{P_{1}}\\ \frac{E_{5}}{P_{2}} & \frac{E_{6}}{P_{2}} & 0 & \frac{E_{3}}{P_{2}} & 0 & 0 & \frac{E_{4}}{P_{2}} & 0 \end{array}\right)$$

The state-space representation transforms the original coupled system into a standard spectral eigenvalue problem. Accordingly, the admissible eigenvalues are obtained from the characteristic equation

(40) det(A−mI)=0.

where I denotes the identity matrix and m represents the eigenvalue parameter governing the attenuation and propagation characteristics of the coupled wave modes inside the semiconductor medium. Expanding Equation (40) yields an eighth-order characteristic polynomial of the form

(41) $$m^{8}+Z_{1}m^{6}+Z_{2}m^{4}+Z_{3}m^{2}+Z_{4}=0$$

where the coefficients $Z_{i}$ are functions of the material parameters, non-local coefficients, thermoelastic coupling constants, and carrier transport quantities. The explicit forms of these coefficients are given in Equation (42).

(42) $$\left.\begin{array}{l} Z_{1}=\frac{-E_{2}E_{4}-E_{3}P_{1}-E_{1}P_{2}-E_{8}P_{2}-E_{10}P_{1}P_{2}-E_{7}P_{1}P_{2}}{P_{1}P_{2}},\\ Z_{2}=E_{1}E_{3}+E_{10}E_{2}E_{4}+E_{2}E_{4}E_{7}+E_{3}E_{8}+E_{2}E_{5}E_{8}+E_{4}E_{9}+E_{10}E_{3}P_{1}+E_{3}E_{7}P_{1}-E_{5}E_{9}P_{1}+E_{1}E_{10}P_{2}\\ +E_{1}E_{7}P_{2}+a_{18}E_{8}P_{2}+E_{10}E_{8}P_{2}+a_{13}a_{18}P_{1}P_{2}+E_{10}E_{7}P_{1}P_{2}/P_{1}P_{2}\\ Z_{3}=(-E_{1}E_{10}E_{3}-a_{13}a_{18}E_{2}E_{4}-E_{1}E_{3}E_{7}-E_{10}E_{2}E_{4}E_{7}-a_{18}E_{3}E_{8}-E_{10}E_{3}E_{8}-E_{10}E_{2}E_{5}E_{8}-a_{18}E_{2}\\ E_{6}E_{8}-a_{18}E_{4}E_{9}-E_{10}E_{4}E_{9}+E_{1}E_{5}E_{9}-a_{13}a_{18}E_{3}P_{1}-E_{10}E_{3}E_{7}P_{1}+E_{10}E_{5}E_{9}P_{1}+a_{18}E_{6}E_{9}P_{1}-a_{13}a_{18}\\ E_{1}P_{2}-E_{1}E_{10}E_{7}P_{2})/P_{1}P_{2}\\ Z_{4}=\frac{a_{13}a_{18}E_{1}E_{3}+E_{1}E_{10}E_{3}E_{7}-E_{1}E_{10}E_{5}E_{9}-a_{18}E_{1}E_{6}E_{9}}{P_{1}P_{2}} \end{array}\right\}$$

The roots of the characteristic polynomial determine the admissible eigenvalues $m_{i}$, which govern the attenuation behavior and spatial propagation of the coupled fields within the semiconductor half-space. Since the present problem is formulated in the semi-infinite domain x≥0, only physically bounded solutions satisfying the regularity condition at infinity are retained. Consequently, the admissible eigenvalues must satisfy $Re(m_{i})>0,$ which guarantees exponentially decaying solutions of the form $e^{-m_{i}x}$ inside the medium. For each eigenvalue $m_{i}$, the corresponding eigenvector is obtained from the algebraic eigenvalue system

(43) $$(A-m_{i}I)q=0.$$

where the eigenvector q is expressed as

(44) $$q=(q_{1},q_{2},q_{3},q_{4},q_{5},q_{6},q_{7},q_{8}{)}^{T}.$$

Expanding Equation (43) produces the associated system of linear algebraic equations governing the eigenvector components

(45) $$\left(-E_{7}+m^{2}\right)\;q_{1}-a_{13}\;q_{2}-E_{8}\;m\;q_{3}-E_{9}\;q_{4}=0$$

(46) $$a_{18}\;q_{1}+\left(-E_{10}+m^{2}\right)\;q_{2}=0$$

(47) $$-\frac{mq_{1}}{P_{1}}-\frac{mq_{2}}{P_{1}}+\left(m^{2}-\frac{E_{1}}{P_{1}}\right)q_{3}+\frac{E_{2}m}{P_{1}}q_{4}=0$$

(48) $$-\frac{E_{5}q_{1}}{P_{2}}-\frac{E_{6}q_{2}}{P_{2}}+\frac{E_{4}}{P_{2}}q_{3}+\left(m^{2}-\frac{E_{3}}{P_{2}}\right)q_{4}=0$$

Because the eigenvalue problem is homogeneous, the eigenvectors are determined up to an arbitrary multiplicative constant. Therefore, a normalization condition is imposed to obtain unique and numerically stable eigenvectors. In the present analysis, the normalization condition is selected such that $q_{1}=1.$ The remaining eigenvector $q_{2},q_{3},q_{4}$ components are subsequently evaluated from the associated algebraic system Equations (45)–(48). Their explicit expressions are presented as

$$\left.\begin{array}{l} q_{2}^{\left(i\right)}=-\;(E_{3}m^{4}P_{1}+E_{1}m^{4}P_{2}+E_{8}m^{4}P_{2}+E_{7}m^{4}P_{1}P_{2}+E_{2}E_{4}m^{4}-E_{3}E_{7}m^{2}P_{1}+E_{5}E_{9}m^{2}P_{1}\\ -E_{1}E_{7}m^{2}P_{2}-E_{1}E_{3}m^{2}-E_{2}E_{4}E_{7}m^{2}-E_{3}E_{8}m^{2}-E_{2}E_{5}E_{8}m^{2}-E_{4}E_{9}m^{2}+E_{1}E_{3}E_{7}-E_{1}E_{5}\\ E_{9}+m^{6}\left(-P_{1}\right)P_{2}/-a_{13}E_{3}m^{2}P_{1}-a_{13}E_{1}m^{2}P_{2}-a_{13}E_{2}E_{4}m^{2}+a_{13}E_{1}E_{3}+a_{13}m^{4}P_{1}P_{2}+E_{8}\\ m^{4}P_{2}+E_{6}E_{9}m^{2}P_{1}-E_{3}E_{8}m^{2}-E_{2}E_{6}E_{8}m^{2}-E_{4}E_{9}m^{2}-E_{1}E_{6}E_{9}) \end{array}\right\},\;\left.\begin{array}{l} q_{3}^{\left(i\right)}=-(-a_{13}E_{3}m-a_{13}E_{2}E_{5}m+a_{13}m^{3}P_{2}-E_{7}m^{3}P_{2}-E_{3}m^{3}-E_{2}E_{6}m^{3}+E_{3}E_{7}m+E_{2}E_{6}\\ E_{7}m-E_{5}E_{9}m+E_{6}E_{9}m+m^{5}P_{2}/a_{13}E_{3}m^{2}P_{1}+a_{13}E_{1}m^{2}P_{2}+a_{13}E_{2}E_{4}m^{2}-a_{13}E_{1}E_{3}-\\ a_{13}m^{4}P_{1}P_{2}-E_{8}m^{4}P_{2}-E_{6}E_{9}m^{2}P_{1}+E_{3}E_{8}m^{2}+E_{2}E_{6}E_{8}m^{2}+E_{4}E_{9}m^{2}+E_{1}E_{6}E_{9} \end{array}\right\},\;\left.\begin{array}{l} q_{4}^{\left(i\right)}=-(a_{13}E_{5}m^{2}P_{1}-a_{13}E_{4}m^{2}-a_{13}E_{1}E_{5}+E_{6}m^{4}P_{1}-E_{4}m^{4}-E_{6}E_{7}m^{2}P_{1}-E_{1}E_{6}m^{2}+\\ E_{4}E_{7}m^{2}+E_{5}E_{8}m^{2}-E_{6}E_{8}m^{2}+E_{1}E_{6}E_{7}/a_{13}E_{3}m^{2}P_{1}+a_{13}E_{1}m^{2}P_{2}+a_{13}E_{2}E_{4}m^{2}-\\ a_{13}E_{1}E_{3}-a_{13}m^{4}P_{1}P_{2}-E_{8}m^{4}P_{2}-E_{6}E_{9}m^{2}P_{1}+E_{3}E_{8}m^{2}+E_{2}E_{6}E_{8}m^{2}+E_{4}E_{9}m^{2}+\\ E_{1}E_{6}E_{9} \end{array}\right\}.$$

Accordingly, the complete analytical solution of the coupled non-local photo-thermoelastic problem can be constructed as a linear superposition of the admissible eigenmodes associated with the retained eigenvalues and their corresponding eigenvectors. The present state-space spectral formulation provides a robust mathematical framework for analyzing the combined effects of nonlocality, anisotropic fiber reinforcement, optoelectronic excitation, and carrier transport on wave propagation characteristics inside semiconductor materials. Since the governing equations form a coupled linear system, the solution can be represented in terms of exponential modes associated with the eigenvalues of the characteristic equation. Accordingly, the transformed field vector is assumed in the form of a linear superposition of independent eigen-solutions. Let $m_{i}\left(i=1,2,3,4\right)$ denote the roots of the characteristic equation and let $q^{\left(i\right)}$ be the corresponding eigenvectors. Then, the general solution vector can be written as

(49) $$V(x)=\sum_{i=1}^{4}C_{i}\;q^{\left(i\right)}e^{-m_{i}x}$$

where $C_{i}$ are arbitrary constants. These constants are not independent physically, but will be evaluated later using the prescribed boundary conditions of the problem. Expanding the vector form into its individual physical quantities, the transformed temperature, carrier density, and displacement components are obtained as linear combinations of the eigenmodes. Hence, each field variable is expressed as a weighted sum of exponential terms governed by the characteristic roots. Therefore, the transformed solutions of the physical fields may be written as follows:

(50) $$T^{*}(x)=\sum_{i=1}^{4}C_{i}\;q_{1}^{\left(i\right)}e^{-m_{i}x}$$

(51) $$N^{*}(x)=\sum_{i=1}^{4}C_{i}\;q_{2}^{\left(i\right)}e^{-m_{i}x}$$

(52) $$u^{*}(x)=\sum_{i=1}^{4}C_{i}\;q_{3}^{\left(i\right)}e^{-m_{i}x}$$

(53) $$v^{*}(x)=\sum_{i=1}^{4}C_{i}\;q_{4}^{\left(i\right)}e^{-m_{i}x}.$$

These expressions represent the complete transformed-domain solution of the problem and constitute the basis for applying the boundary conditions and deriving the final closed-form expressions of the physical fields. The transformed stress components associated with Equations (28)–(30) can also be expressed in the same exponential modal representation adopted for the transformed field variables. Since the governing system is linear, the stress fields are represented as superpositions of exponentially decaying eigenmodes corresponding to the characteristic roots $m_{i}$. Using the previously obtained transformed field solutions given in Equations (50)–(53), the transformed stress components are assumed in the forms

(54) $$\sigma_{xx}^{*}(x)=\sum_{i=1}^{4}C_{i}\;q_{5}^{\left(i\right)}e^{-m_{i}x}$$

(55) $$\sigma_{yy}^{*}(x)=\sum_{i=1}^{4}C_{i}\;q_{6}^{\left(i\right)}e^{-m_{i}x}$$

(56) $$\sigma_{xy}^{*}(x)=\sum_{i=1}^{4}C_{i}\;q_{7}^{\left(i\right)}e^{-m_{i}x}$$

where $q_{5}^{\left(i\right)}$, $q_{6}^{\left(i\right)}$, and $q_{7}^{\left(i\right)}$ denote the modal coefficients corresponding to the transformed stress components $\sigma_{xx}^{*}$, $\sigma_{yy}^{*}$, and $\sigma_{xy}^{*}$, respectively. Substituting these modal solutions into the constitutive relations (28)–(30) and equating the coefficients of the independent exponential terms yield the following expressions for the stress modal coefficients:

(57) $$q_{5}^{\left(i\right)}=\frac{m_{i}q_{3}^{\left(i\right)}-iaa_{1}q_{4}^{\left(i\right)}+1+q_{2}^{\left(i\right)}}{\xi^{2}m_{i}^{2}-(1+a^{2}\xi^{2})}$$

(58) $$q_{6}^{\left(i\right)}=\frac{a_{1}m_{i}q_{3}^{\left(i\right)}-iaa_{2}q_{4}^{\left(i\right)}+a_{3}+a_{4}q_{2}^{\left(i\right)}}{\xi^{2}m_{i}^{2}-(1+a^{2}\xi^{2})}$$

(59) $$q_{7}^{\left(i\right)}=\frac{-iaa_{5}q_{3}^{\left(i\right)}+a_{5}m_{i}q_{4}^{\left(i\right)}}{\xi^{2}m_{i}^{2}-(1+a^{2}\xi^{2})}$$

Therefore, all transformed stress components are completely determined in terms of the characteristic roots and the associated eigenvector components of the coupled rotating anisotropic semiconductor system.

## 6. Boundary Conditions and Computational Procedure

To determine the unknown constants $C_{i}$ appearing in the general modal solutions, the prescribed boundary conditions are imposed at the surface x=0. By substituting the obtained analytical solutions into the governing boundary conditions, a coupled linear algebraic system is generated for the unknown coefficients $C_{i}$. The thermal, carrier density, displacement, and stress boundary conditions are expressed in terms of the eigenmode expansions obtained previously. Consequently, the resulting equations can be assembled into the compact matrix form BC=D, where B denotes the coefficient matrix constructed from the eigenvector components evaluated at the boundary, $C=(C_{1},C_{2},C_{3},C_{4}{)}^{T}$ represents the vector of unknown modal constants, and D is the right-hand-side vector determined from the imposed physical boundary conditions. The resulting linear system was solved computationally using Mathematica, where the coefficient matrix and forcing vector were generated explicitly and the unknown constants were obtained using the LinearSolve routine. These constants were then substituted back into the general solutions to obtain the complete expressions of the physical fields within the semiconductor medium. The imposed boundary conditions are summarized as follows.

Temperature Constraint

(60) $$T^{*}(0,y,t)=\theta_{0}e^{\omega t+iay}.$$

This condition represents the harmonically varying surface temperature generated by periodic optical excitation. The parameter $\theta_{0}$ characterizes the amplitude of the applied thermal loading.

Carrier Density Constraint

(61) $$N^{*}(0,y,t)=N_{0}e^{\omega t+iay}$$

This boundary condition describes the photo-generated carrier density induced by optical illumination at the surface of the semiconductor medium.

Displacement Constraint

(62) $$v^{*}(0,y,t)=0$$

This condition indicates that the surface is mechanically constrained in the transverse direction, implying the absence of displacement along the v-direction at the boundary.

Shear Stress Constraint

(63) $$\sigma_{xy}(0,y,t)=0.$$

**Figure 2:**
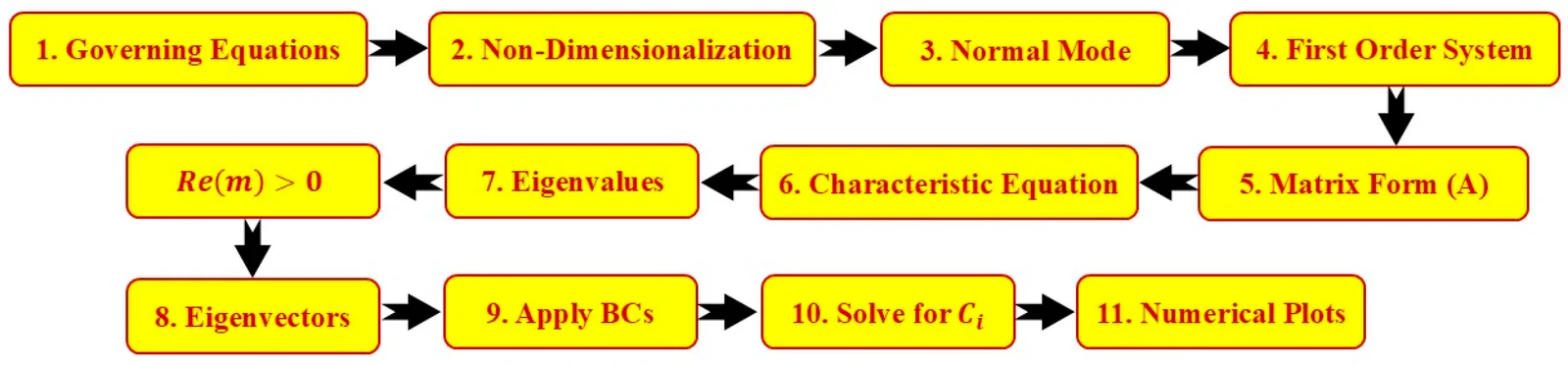
Computational workflow of the proposed method from the governing equations to the numerical results.

This condition corresponds to a traction-free surface with respect to shear stress, ensuring the absence of tangential surface forces. In addition to the boundary conditions imposed at the surface x=0, the regularity condition at infinity is also required to guarantee physically bounded solutions inside the semi-infinite medium. Therefore, $T^{*},\;N^{*},\;u^{*},\;v^{*}\to 0\;\mathrm{as}\;x\to \infty .$ These conditions ensure the physical admissibility and stability of the obtained analytical solutions. The combination of the mechanical boundary conditions $v^{*}\left(0,y,t\right)=0$ and $\sigma_{xy}\left(0,y,t\right)=0$ represents a mechanically constrained surface that is restrained against transverse displacement while remaining free of tangential shear traction. This configuration models a guided boundary, where the normal mechanical constraint suppresses rigid-body motion in the transverse direction without introducing tangential frictional forces. Such a combination is frequently adopted in generalized thermoelastic analyses of semi-infinite media to isolate the coupled thermo-mechanical and photo-induced responses from unnecessary boundary-induced shear effects, thereby providing a physically meaningful and mathematically well-posed boundary-value problem [32]. Alternative boundary conditions, such as fully traction-free or fully clamped surfaces, may also be employed to represent different physical configurations. However, the present guided-boundary configuration is adopted because it provides an idealized representation of a mechanically constrained semiconductor surface under optical excitation while minimizing boundary-induced shear effects and enabling a clear investigation of the coupled photo-thermoelastic response. The overall computational procedure adopted in the present work, from the governing equations to the numerical results, is summarized in Figure 2. The selection of these guided boundary conditions is also motivated by the need to maintain a well-posed coupled boundary-value problem within the adopted eigenvalue framework. This configuration provides a physically consistent representation of a constrained semiconductor surface while allowing the coupled thermoelastic and photo-induced responses to be examined without introducing additional boundary-induced shear effects. Accordingly, the guided boundary configuration is retained as the reference mechanical boundary condition throughout the present analysis.

## 7. Numerical Accuracy and Stability of the Eigenvalue Method

The present numerical results are obtained from an exact analytical solution based on the eigenvalue–eigenvector formulation rather than from an approximate numerical discretization. The characteristic roots are determined from the corresponding characteristic equation, while only the physically admissible eigenvalues satisfying $Re\left(m\right)<0$ are retained to satisfy the regularity condition at infinity. Consequently, the obtained field distributions are numerically stable and physically bounded. Since the present investigation does not rely on experimental measurements or stochastic simulations, uncertainty bounds or error bars are not applicable. The accuracy of the results is further supported by the limiting-case validations presented in the following section.

## 8. Model Validation Through Limiting Cases

### 8.1. Limiting Isotropic Case

To further verify the mathematical consistency and generality of the proposed anisotropic fiber-reinforced formulation, the limiting isotropic case is considered. In this limit, the directional dependence introduced by the reinforcing fibers disappears, and the material properties become identical in all directions. Accordingly, the anisotropic constitutive coefficients reduce to their isotropic counterparts according to

$$A_{11}=\lambda +2\mu ,A_{12}=\lambda ,A_{13}=\lambda +2\mu ,A_{14}=\mu ,\beta_{11}=\beta_{22}=\gamma_{T},\eta_{11}=\eta_{22}=\gamma_{N},K_{11}=K_{22}=K.$$

Consequently, the constitutive relations reduce to

(64) $$\left(1-\xi^{2}\nabla^{2}\right)\sigma_{xx}=\left(\lambda +2\mu \right)\frac{\partial u}{\partial x}+\lambda \frac{\partial v}{\partial y}-\gamma_{T}T-\gamma_{N}N$$

(65) $$\left(1-\xi^{2}\nabla^{2}\right)\sigma_{yy}=\left(\lambda +2\mu \right)\frac{\partial v}{\partial y}+\lambda \frac{\partial u}{\partial x}-\gamma_{T}T-\gamma_{N}N$$

(66) $$\left(1-\xi^{2}\nabla^{2}\right)\sigma_{xy}=\mu \left(\frac{\partial u}{\partial y}+\frac{\partial v}{\partial x}\right)$$

Here, $\gamma_{T}=\left(3\lambda +2\mu \right)\alpha_{t},\gamma_{N}=\left(3\lambda +2\mu \right)d_{n}$ denote the effective thermoelastic and carrier-elastic coupling coefficients of the isotropic medium, respectively. These coefficients are recovered from the anisotropic formulation by imposing the isotropic limiting conditions, under which the directional material properties become identical in all directions. Consequently, the thermoelastic and carrier-elastic coupling coefficients satisfy $\beta_{11}=\beta_{22}=\gamma_{T}$ and $\eta_{11}=\eta_{22}=\gamma_{N}$, while the effective thermal conductivities reduce to $K_{11}=K_{22}=K$. Similarly, the heat-conduction equation reduces to its isotropic form as

(67) $$K\nabla^{2}T+\frac{E_{g}}{\tau }N=\rho C_{e}\frac{\partial T}{\partial t}+T_{0}\gamma_{T}\left(\frac{\partial u}{\partial x}+\frac{\partial v}{\partial y}\right)$$

Therefore, the complete governing equations, including the constitutive relations, the heat-conduction equation, and the equations of motion, reduce exactly to the classical isotropic non-local photo-thermoelastic model reported in [32,33,34]. This limiting case further confirms the mathematical consistency and generality of the proposed anisotropic fiber-reinforced formulation.

### 8.2. Limiting Local Case

To further validate the proposed non-local formulation, a direct mathematical comparison is performed with the corresponding local anisotropic fiber-reinforced photo-thermoelastic model reported in Ref. [16]. The comparison is made at the level of the constitutive relations, equations of motion, heat-conduction equation, carrier-density equation, and the resulting coefficient matrix.

In the present study, the non-local constitutive relations are given by Equations (3)–(5):

$$\left(1-\xi^{2}\nabla^{2}\right)\sigma_{xx}=A_{11}\frac{\partial u}{\partial x}+A_{12}\frac{\partial v}{\partial y}-\beta_{11}T-\eta_{11}N,$$

$$\left(1-\xi^{2}\nabla^{2}\right)\sigma_{yy}=A_{12}\frac{\partial u}{\partial x}+A_{13}\frac{\partial v}{\partial y}-\beta_{22}T-\eta_{22}N,$$

$$\;\left(1-\xi^{2}\nabla^{2}\right)\sigma_{xy}=A_{14}\left(\frac{\partial u}{\partial y}+\frac{\partial v}{\partial x}\right).$$

The corresponding constitutive relations in Ref. [16], given by Equations (3)–(5), are

$$\sigma_{xx}=A_{11}\frac{\partial u}{\partial x}+A_{12}\frac{\partial v}{\partial y}-\beta_{11}T-\eta_{11}N,$$

$$\sigma_{yy}=A_{12}\frac{\partial u}{\partial x}+A_{13}\frac{\partial v}{\partial y}-\beta_{22}T-\eta_{22}N,$$

$$\;\sigma_{xy}=A_{14}\left(\frac{\partial u}{\partial y}+\frac{\partial v}{\partial x}\right).$$

Thus, the fundamental constitutive structure is identical, while the present formulation introduces the additional non-local operator $1-\xi^{2}\nabla^{2}$ on the stress components.

A similar distinction is observed in the equations of motion. The present non-local formulation is governed by Equations (10) and (11)

$$\left(1-\xi^{2}\nabla^{2}\right)\rho u_{tt}=A_{11}u_{xx}+A_{14}u_{yy}+\left(A_{12}+A_{14}\right)v_{xy}-\beta_{11}T_{x}-\eta_{11}N_{x},$$

$$\;\left(1-\xi^{2}\nabla^{2}\right)\rho v_{tt}=\left(A_{12}+A_{14}\right)u_{xy}+A_{14}v_{xx}+A_{13}v_{yy}-\beta_{22}T_{y}-\eta_{22}N_{y}.$$

In contrast, the corresponding equations in Ref. [16], Equations (10) and (11), are

$$A_{11}u_{xx}+A_{14}u_{yy}+\left(A_{12}+A_{14}\right)v_{xy}-\beta_{11}T_{x}-\eta_{11}N_{x}=\rho u_{tt},$$

$$\left(A_{12}+A_{14}\right)u_{xy}+A_{14}v_{xx}+A_{13}v_{yy}-\beta_{22}T_{y}-\eta_{22}N_{y}=\rho v_{tt}.$$

Therefore, the difference between the two formulations at the mechanical level is explicitly associated with the non-local operator in Equations (10) and (11), which is absent from the local model of Ref. [16].

For the thermal and carrier-density fields, however, the governing equations retain the same mathematical form. The present thermal equation, Equation (13), is

$$K_{11}T_{xx}+K_{22}T_{yy}+\frac{E_{g}}{\tau }N=\rho C_{E}T_{t}+T_{0}\left(\beta_{11}u_{xt}+\beta_{22}v_{yt}\right).$$

whereas the corresponding equation in Ref. [16], Equation (13), has the same form. Likewise, the carrier-density equation of the present study

$$\;\frac{\partial N}{\partial t}=D_{E}\nabla^{2}N-\frac{N}{\tau }+\kappa T.$$

corresponds directly to Equation (14) of Ref. [16]. Hence, the introduction of non-locality modifies the mechanical and constitutive parts of the formulation while preserving the mathematical structure of the thermal and carrier-density equations.

At the eigenvalue level, Ref. [16] transforms its coupled local system into a first-order matrix system represented by the coefficient matrix given in Equation (38):

$$A=\left(\begin{array}{cccccccc} 0 & 0 & 0 & 0 & 1 & 0 & 0 & 0\\ 0 & 0 & 0 & 0 & 0 & 1 & 0 & 0\\ 0 & 0 & 0 & 0 & 0 & 0 & 1 & 0\\ 0 & 0 & 0 & 0 & 0 & 0 & 0 & 1\\ E_{7} & -a_{13} & 0 & E_{9} & 0 & 0 & E_{8} & 0\\ -a_{18} & E_{10} & 0 & 0 & 0 & 0 & 0 & 0\\ 0 & 0 & E_{1} & 0 & 1 & 1 & 0 & -E_{2}\\ E_{5} & E_{6} & 0 & E_{3} & 0 & 0 & -E_{4} & 0 \end{array}\right).$$

In the present study, the corresponding coefficient matrix A takes the form in Equation (39):

$$A=\left(\begin{array}{cccccccc} 0 & 0 & 0 & 0 & 1 & 0 & 0 & 0\\ 0 & 0 & 0 & 0 & 0 & 1 & 0 & 0\\ 0 & 0 & 0 & 0 & 0 & 0 & 1 & 0\\ 0 & 0 & 0 & 0 & 0 & 0 & 0 & 1\\ E_{7} & a_{13} & 0 & E_{9} & 0 & 0 & E_{8} & 0\\ -a_{18} & E_{10} & 0 & 0 & 0 & 0 & 0 & 0\\ 0 & 0 & \frac{E_{1}}{P_{1}} & 0 & \frac{1}{P_{1}} & \frac{1}{P_{1}} & 0 & -\frac{E_{2}}{P_{1}}\\ \frac{E_{5}}{P_{2}} & \frac{E_{6}}{P_{2}} & 0 & \frac{E_{3}}{P_{2}} & 0 & 0 & \frac{E_{4}}{P_{2}} & 0 \end{array}\right).$$

Comparing Equation (39) with Equation (38) shows that the present coefficient matrix contains additional $P_{1}$- and $P_{2}$-dependent terms in the lower rows resulting directly from the non-local formulation. These terms modify the coefficients of the eigenvalue system and therefore distinguish the proposed non-local model from the local formulation of Ref. [16]. Nevertheless, when the non-local parameter is set to zero, ξ=0, the non-local operators reduce to unity ($P_{1}\to 1$ and $P_{2}\to 1$), and the $P_{1}$- and $P_{2}$-dependent modifications reduce to their local-limit forms. Consequently, the resulting matrix in Equation (39) recovers the exact fundamental coupling structure among the displacement, temperature, and carrier-density variables as the coefficient matrix in Equation (38) of Ref. [16].

Overall, this comparison demonstrates that the present model is a genuine non-local extension of the established formulation in Ref. [16], rather than a fundamentally different governing framework. The constitutive and mechanical equations acquire the non-local operator, whereas the thermal and carrier-density equations retain their original structure. Moreover, the local limit ξ=0 consistently recovers the corresponding local governing equations and eigenvalue structure reported in Ref. [16], thereby providing a direct mathematical verification of the proposed formulation.

As an additional quantitative verification, the temperature field obtained from the present formulation in the local limit (ξ=0) is compared with the corresponding results reported in [43]. For consistency, the comparison is performed at t=0.9 using identical spatial positions. As shown in Table 1, the two sets of temperature values exhibit very close agreement, with the relative difference remaining below 0.11% over the selected points. This close quantitative agreement provides additional support for the correctness of the present formulation and complements the analytical limiting-case verification.

## 9. Numerical Results and Discussion

In this section, the obtained analytical solutions are evaluated numerically to investigate the coupled photo-thermoelastic response of the anisotropic semiconductor medium within the framework of the non-local theory and under the effect of fiber reinforcement. The numerical computations are performed using the physical properties of silicon listed in Table 2, where all governing equations and field quantities are considered in non-dimensional form. Accordingly, unless otherwise stated, all spatial coordinates and field variables presented in the following figures are expressed in dimensionless form. The present model combines three important physical mechanisms. The present model combines three important physical mechanisms, namely photothermal excitation, non-local elasticity, and fiber reinforcement, which together provide a more realistic description of the behavior of modern semiconductor materials at micro- and nanoscales. The inclusion of non-local effects allows the model to capture the influence of small-scale interactions that cannot be described accurately by classical thermoelastic theories, while the fiber reinforcement improves the mechanical stability and directional stiffness of the medium. Such coupled models have important applications in semiconductor devices, nano-electromechanical systems (NEMS), optoelectronic structures, laser-heated materials, and advanced composite materials subjected to thermal and optical loading. The derived analytical expressions are implemented computationally using Mathematica to evaluate the spatial distributions of the thermal, carrier density, displacement, and stress fields throughout the semiconductor medium. Special attention is devoted to studying the influence of the non-local parameter ξ and examining the differences between the reinforced and non-reinforced cases. The numerical profiles of the temperature T, carrier density N, displacement components u and v, and stress components $\sigma_{xx}$, $\sigma_{yy}$, and $\sigma_{xy}$ are presented graphically to illustrate the effects of the coupled physical parameters on the propagation and attenuation behavior of the considered fields. The following subsections discuss the numerical results obtained and provide a physical interpretation of the observed behavior of the coupled photo-thermoelastic fields.

## 10. Effect of the Non-Local Parameter

**Figure 3:**
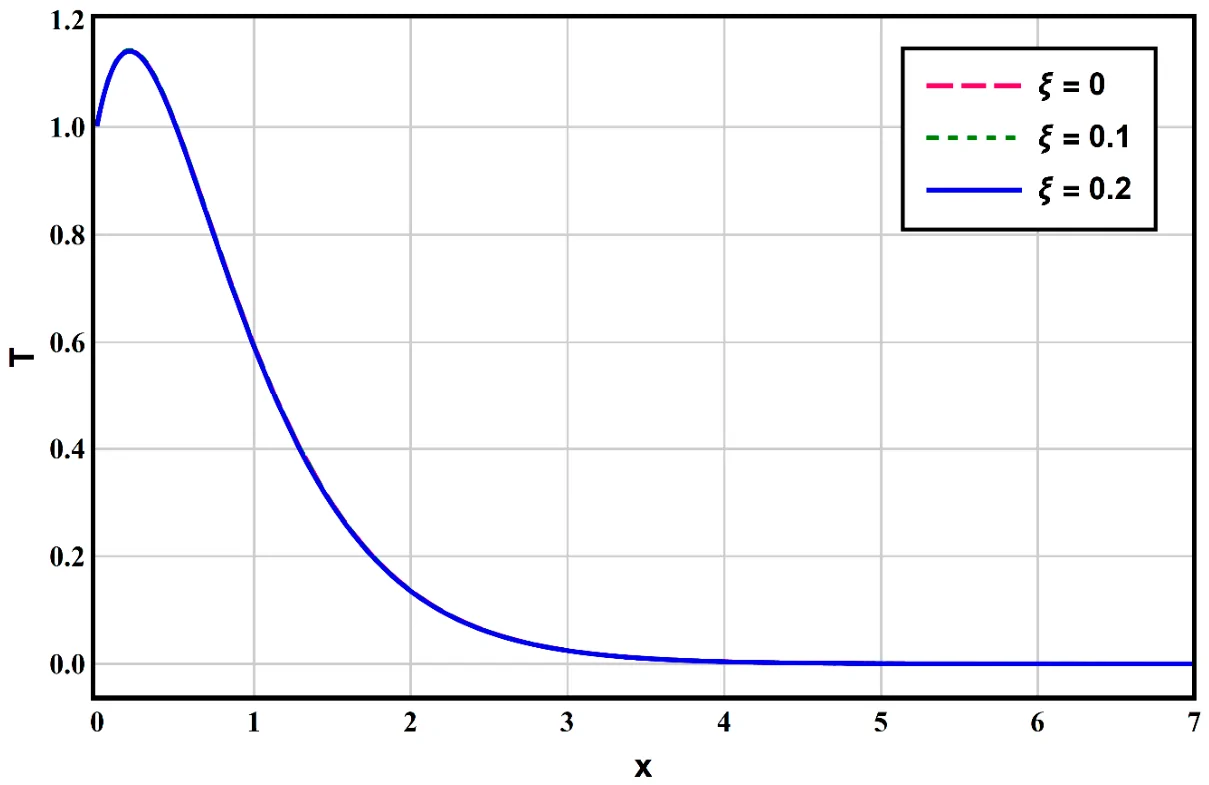
Variation in the non-dimensional temperature distribution T along the spatial coordinate x for different values of the non-local parameter ξ, illustrating the influence of nonlocality on the photo-thermoelastic response of the fiber-reinforced anisotropic semiconductor medium.

**Figure 4:**
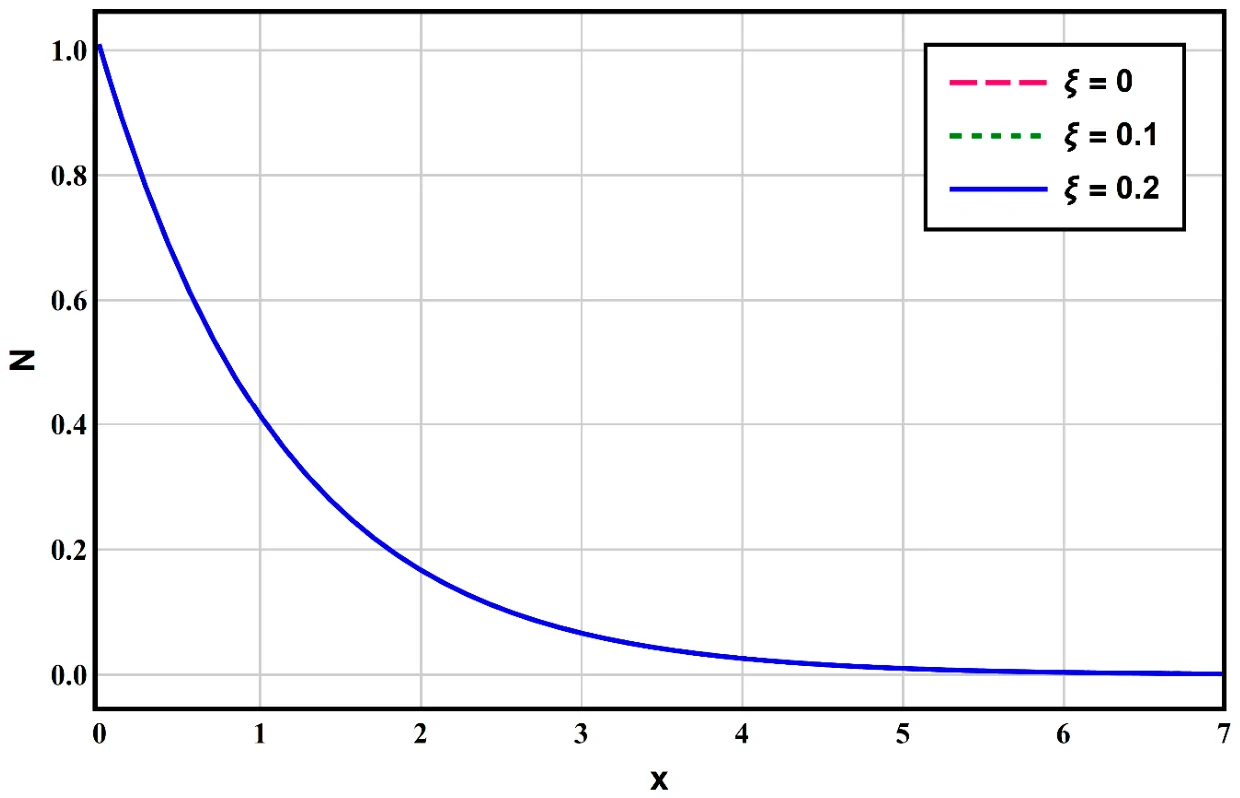
Variation in the non-dimensional carrier-density distribution N along the spatial coordinate x for different values of the non-local parameter ξ, demonstrating the influence of nonlocality on carrier transport in the fiber-reinforced anisotropic semiconductor medium.

**Figure 5:**
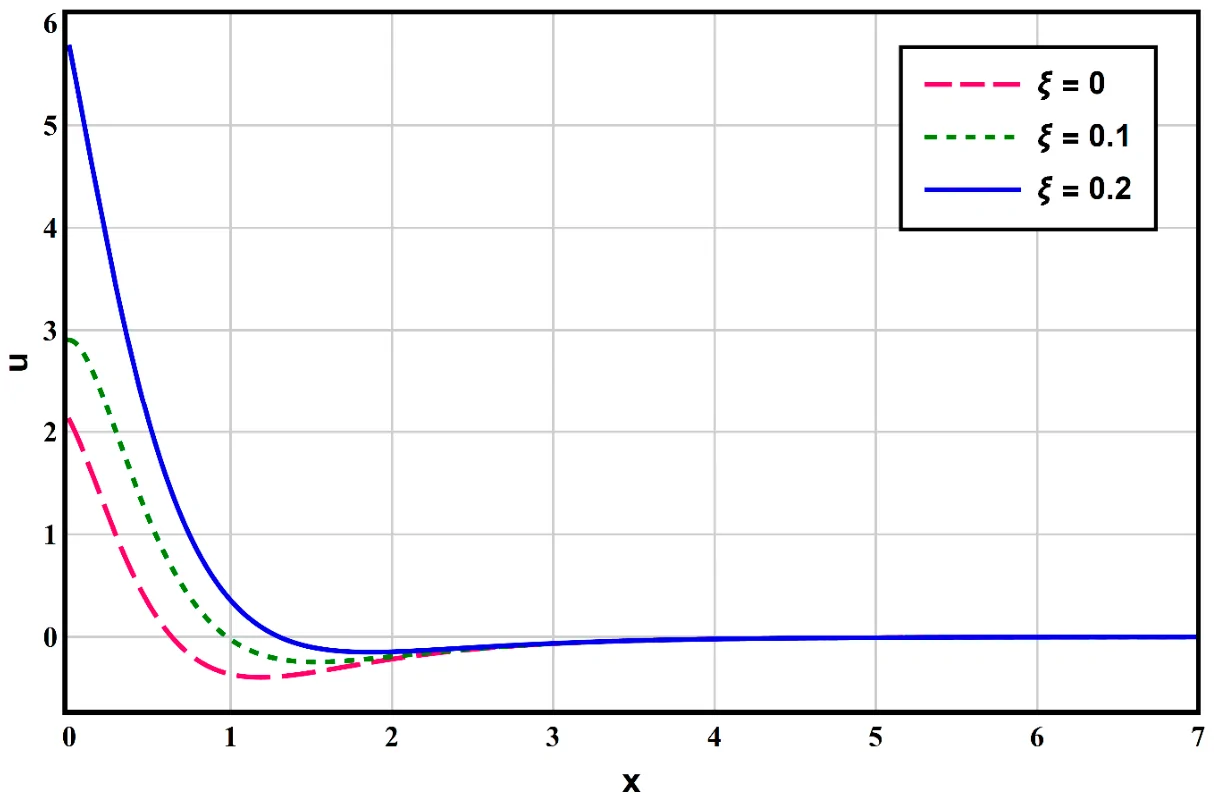
Spatial variation in the non-dimensional longitudinal displacement u under different values of the non-local parameter ξ, illustrating the effect of nonlocality on the elastic response of the fiber-reinforced anisotropic semiconductor medium.

**Figure 6:**
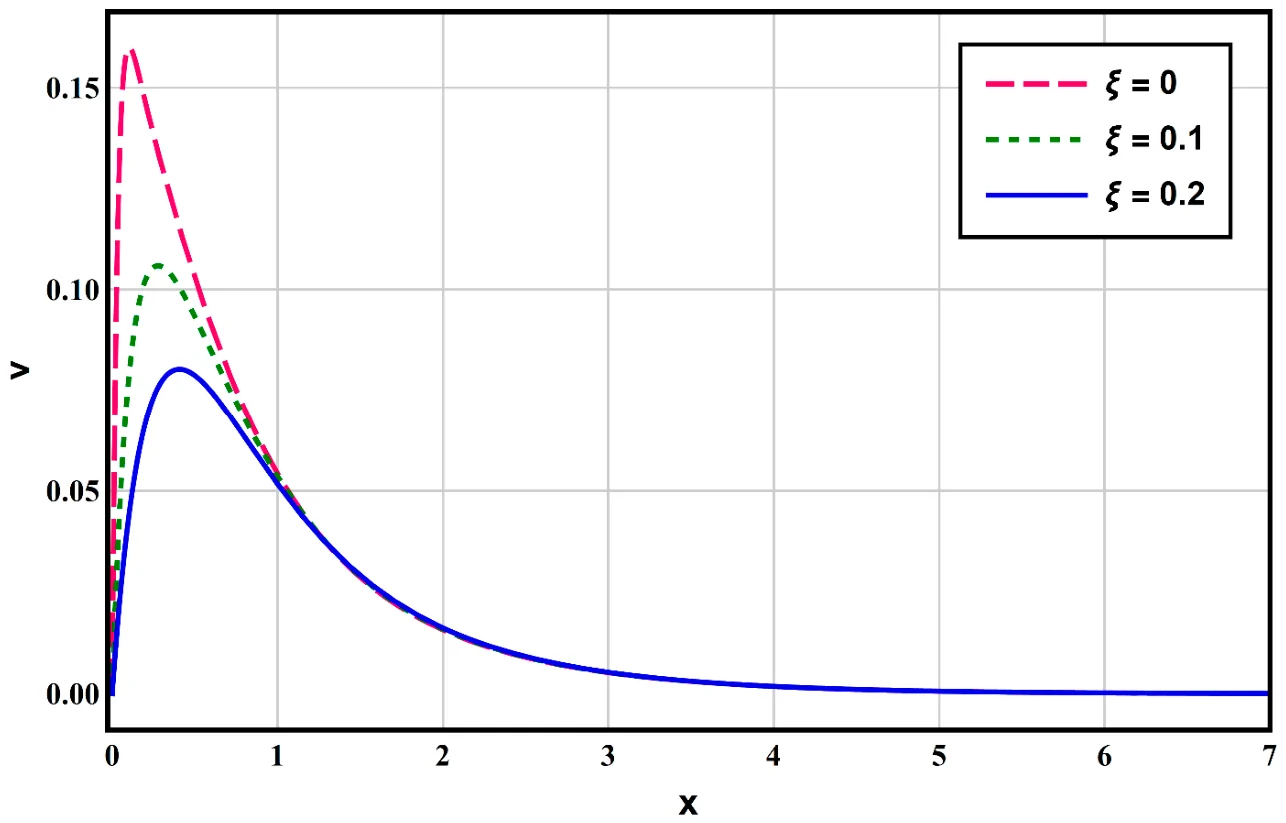
Distribution of the non-dimensional transverse displacement v along the spatial coordinate x for various values of the non-local parameter ξ, highlighting the influence of size-dependent effects on the transverse deformation of the fiber-reinforced anisotropic semiconductor medium.

**Figure 7:**
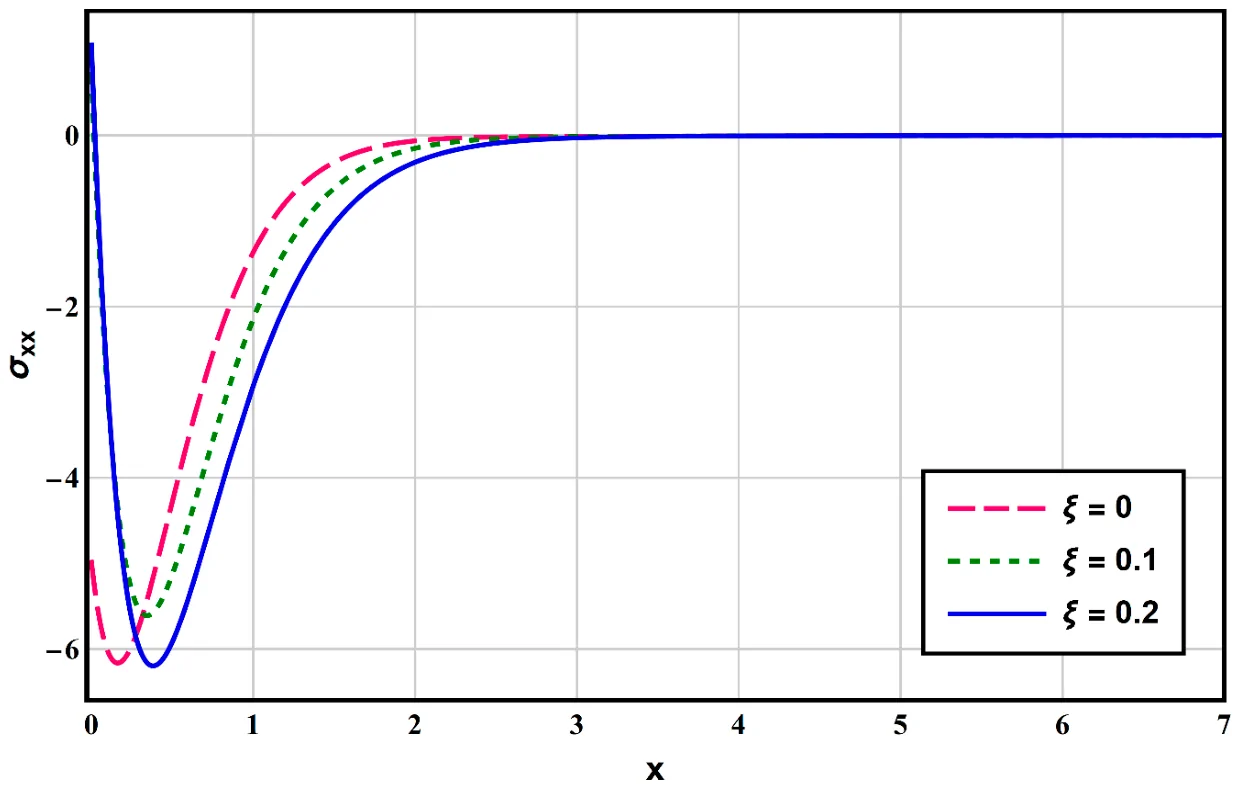
Variation in the non-dimensional normal stress component $\sigma_{xx}$ with the spatial coordinate x for different values of the non-local parameter ξ, showing the influence of nonlocality on the axial stress response of the fiber-reinforced anisotropic semiconductor medium.

**Figure 8:**
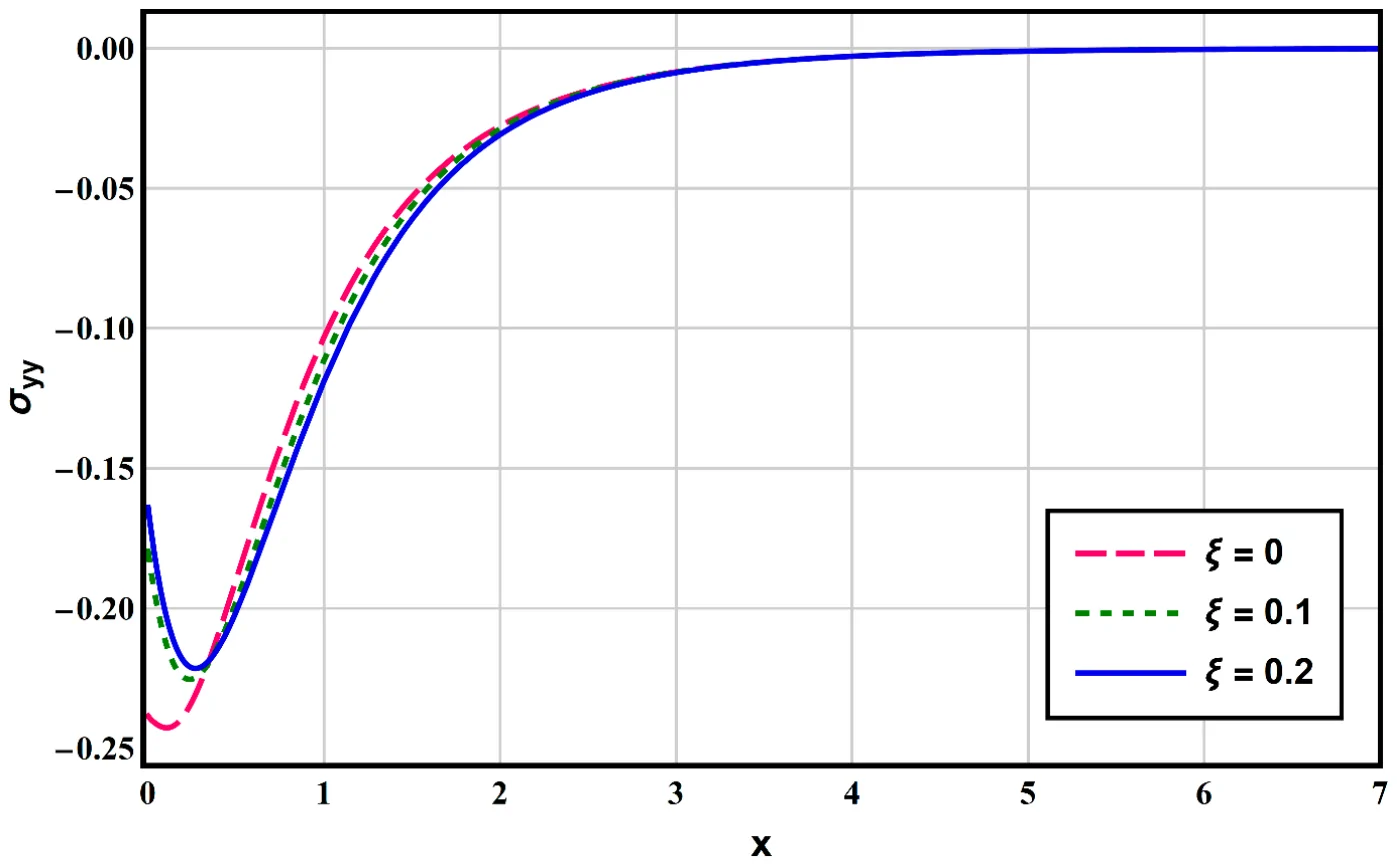
Variation in the non-dimensional normal stress component $\sigma_{yy}$ under different values of the non-local parameter ξ, illustrating the effect of non-local elasticity on the transverse stress distribution within the fiber-reinforced anisotropic semiconductor medium.

**Figure 9:**
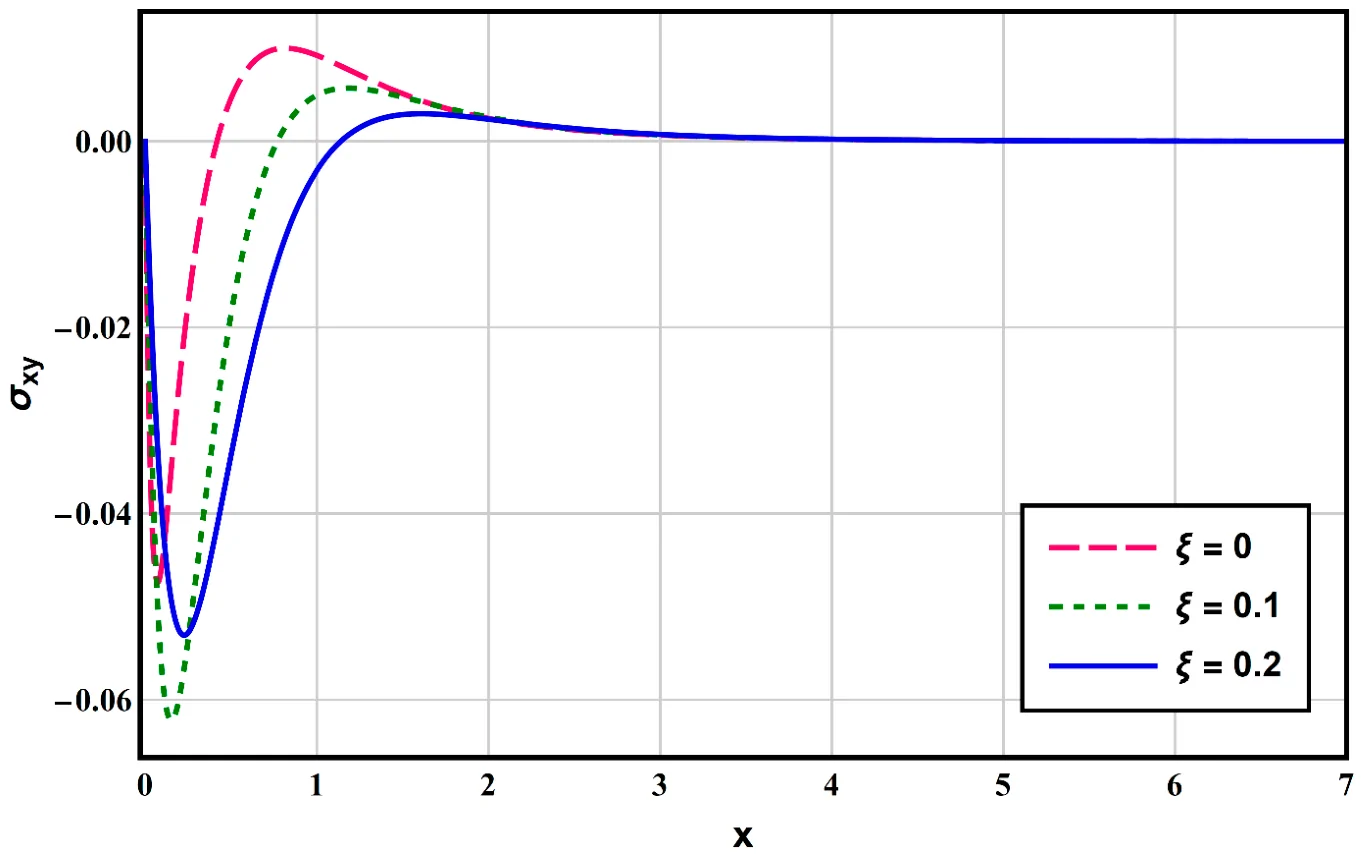
Distribution of the non-dimensional shear stress component $\sigma_{xy}$ along the spatial coordinate x for various values of the non-local parameter ξ, demonstrating the influence of nonlocality on the shear stress behavior of the fiber-reinforced anisotropic semiconductor medium.

In this subsection, the effect of the non-local parameter ξ on the coupled photo-thermoelastic behavior of the fiber-reinforced semiconductor medium is discussed in detail. The numerical computations are performed for three different values of the non-local parameter, namely ξ=0, 0.1, and 0.2, to examine the influence of small-scale interactions on the thermal, elastic, and carrier-density fields. The obtained graphical distributions are presented in Figure 3, Figure 4, Figure 5, Figure 6, Figure 7, Figure 8 and Figure 9 for the temperature T, carrier density N, displacement components u and v, and stress components $\sigma_{xx}$, $\sigma_{yy}$, and $\sigma_{xy}$. It is clear from all figures that the considered field quantities satisfy the regularity and boundedness conditions, where all distributions decay gradually with increasing distance from the illuminated surface. This behavior confirms the physical consistency of the obtained analytical solutions within the semi-infinite semiconductor medium. Furthermore, the influence of the non-local parameter is mainly concentrated near the boundary region, where the coupling between the photothermal and elastic effects becomes dominant. Figure 3 illustrates the variation in the temperature distribution T with the spatial coordinate x. The temperature initially increases near the illuminated boundary due to the absorption of optical energy and the resulting photothermal excitation. After reaching a maximum value, the temperature decreases rapidly as the distance increases because of thermal diffusion inside the medium. It is observed that increasing the non-local parameter slightly modifies the thermal peak and enhances the attenuation behavior. This indicates that the non-local interactions influence the heat transfer mechanism by redistributing the thermal energy over neighboring material particles. To further quantify the weak sensitivity of the temperature field to the non-local parameter, the maximum absolute differences between the temperature profiles were evaluated over 0 ≤ x ≤ 7. The maximum differences were 1.06305 × 10^−3^ between ξ = 0 and ξ = 0.1, 2.07671 × 10^−3^ between ξ = 0.1 and ξ = 0.2, and 2.76028 × 10^−3^ between ξ = 0 and ξ = 0.2. The maximum relative variation between the limiting cases was approximately 1.02%. These small variations quantitatively confirm that the temperature field is only weakly sensitive to the considered range of the non-local parameter, consistent with the nearly overlapping curves shown in Figure 3. Figure 4 represents the carrier density distribution N. The carrier density decreases monotonically with increasing distance from the boundary due to carrier recombination and diffusion processes inside the semiconductor material. The increase in the non-local parameter causes a slight reduction in the penetration depth of the carrier-density field, indicating that the non-local interactions suppress the propagation of carrier waves inside the medium. This behavior reflects the influence of the internal length-scale effect introduced by the non-local theory. Similarly, the carrier-density field exhibits an even weaker sensitivity to the non-local parameter. The maximum absolute differences between the profiles for ξ = 0, 0.1, and 0.2 are 2.19424 × 10 ^−12^, 9.81215 × 10 ^−13^, and 1.21692 × 10 ^−12^, respectively, over 0 ≤ x ≤ 7. These extremely small variations quantitatively confirm the near-independence of the carrier-density distribution from the considered non-local parameter range. The longitudinal displacement component u is presented in Figure 5. The displacement exhibits an attenuating wave-like behavior resulting from the strong coupling between thermal expansion and elastic deformation. It is observed that increasing the non-local parameter significantly increases the magnitude of the displacement near the surface region. Moreover, the locations of the extrema are shifted slightly as ξ increases. This indicates that the non-local interactions strongly affect the elastic wave propagation and modify the mechanical response of the medium. Figure 6 shows the variation in the transverse displacement component v. The displacement initially increases from zero, reaches a maximum value near the boundary, and then decays asymptotically toward zero. Increasing the non-local parameter decreases the peak amplitude of the transverse displacement and accelerates its attenuation inside the medium. This behavior demonstrates that the non-local effect contributes to improving the structural stability and reducing excessive transverse deformation. The behavior of the normal stress component $\sigma_{xx}$ is illustrated in Figure 7. The stress field initially decreases sharply near the boundary and reaches a compressive minimum value before approaching zero gradually for large values of x. Increasing the non-local parameter increases the magnitude of the compressive stress and modifies the stress concentration region. This behavior indicates that the non-local interactions enhance the internal resistance of the medium against deformation and alter the stress redistribution mechanism throughout the semiconductor structure. Figure 8 presents the normal stress component $\sigma_{yy}$. Like $\sigma_{xx}$, the stress field exhibits a compressive nature near the boundary and then decays gradually toward the equilibrium state. The increase in the non-local parameter slightly smooths the stress profile and modifies the stress amplitude near the surface region. Such smoothing behavior is one of the characteristic features of non-local elasticity theory, where the stress state at a point depends on the surrounding material interactions rather than purely local deformation. Figure 9 describes the variation in the shear stress component $\sigma_{xy}$. The shear stress exhibits an oscillatory-decaying behavior characterized by an initial negative peak followed by a gradual return toward zero. Increasing the non-local parameter noticeably changes both the amplitude and attenuation rate of the shear stress field. This result indicates that the non-local interactions strongly influence the propagation of coupled elastic disturbances and redistribute the internal shear forces inside the medium.

## 11. Physical Interpretation

From the physical viewpoint, the obtained behavior can be explained through the fundamental principles of non-local elasticity theory. In classical local elasticity, the stress at a given material point depends only on the strain at the same point. However, in the non-local theory, the stress field depends on the collective interaction of neighboring particles within a finite internal length scale. Consequently, increasing the non-local parameter ξ enhances the interaction range between neighboring particles and modifies the propagation characteristics of the coupled fields. The observed increase in the compressive stress magnitudes and the modifications in displacement amplitudes are direct consequences of the stronger internal interactions generated by the non-local effect. These interactions redistribute the mechanical energy throughout the medium and produce smoother stress variations compared with the classical local case. Similarly, the changes observed in the temperature and carrier-density fields indicate that non-locality slightly alters the diffusion and transport processes inside the semiconductor material. Mathematically, the influence of the non-local parameter appears explicitly through the terms containing $\xi^{2}$ in the governing equations and constitutive relations. These additional terms modify the characteristic roots $m_{i}$, which control the attenuation and propagation behavior of the field variables. Consequently, changing the value of ξ alters the eigenvalue structure of the coupled system and leads to the different numerical distributions observed in the graphical results. Moreover, the coupling between photothermal excitation and fiber reinforcement plays an important role in shaping the overall behavior of the medium. The optical excitation generates thermal and carrier waves inside the semiconductor material, while the reinforcing fibers enhance the directional stiffness and mechanical stability of the structure. The interaction between these physical mechanisms and the non-local effect produces the coupled thermoelastic behavior demonstrated in the presented numerical results. In the present parametric study, the non-local parameter was selected as ξ=0, 0.1, and 0.2 to systematically investigate the influence of nonlocality on the coupled response of the silicon medium. The case ξ=0 represents the classical local elasticity model and serves as the reference configuration, whereas ξ=0.1 and 0.2 correspond to moderate non-local effects that allow the gradual influence of non-local interactions to be clearly identified without masking the overall physical behavior. Physically, the non-local parameter is associated with the internal material length scale and reflects the influence of the silicon microstructure, where long-range interactions among neighboring particles become significant. Therefore, increasing ξ enhances the contribution of microstructural effects, leading to the variations observed in the thermoelastic and photocarrier responses. From a quantitative viewpoint, the influence of the non-local parameter becomes more pronounced in the displacement and stress fields than in the thermal and carrier-density distributions. As ξ increases from 0 to 0.2, the peak longitudinal displacement increases by approximately 160%, whereas the peak transverse displacement decreases by nearly 50%. The maximum compressive normal stress $\sigma_{xx}$ exhibits an increase in magnitude of about 10%, while the peak shear stress $\sigma_{xy}$ changes by approximately 15%. In contrast, the temperature and carrier-density distributions remain only slightly affected by the selected non-local parameter values, indicating that the principal role of nonlocality in the present model is to modify the mechanical response rather than the thermal or carrier transport fields. These quantitative observations are fully consistent with the physical interpretation of the non-local theory, where increasing ξ enhances long-range particle interactions and strengthens the microstructural influence on the coupled photo-thermoelastic response. It is worth noting that, in the framework of non-local elasticity theories applied to micro-/nano-scale semiconductor and composite structures, the internal characteristic length scale associated with the non-local parameter is typically reported to lie within the sub-nanometer to few-nanometer range. The adopted values (*ξ* = 0.1, 0.2) are therefore consistent with this physically established order of magnitude for silicon-based microstructures.

## 12. Comparison Between Fiber-Reinforced and Non-Reinforced Media

**Figure 10:**
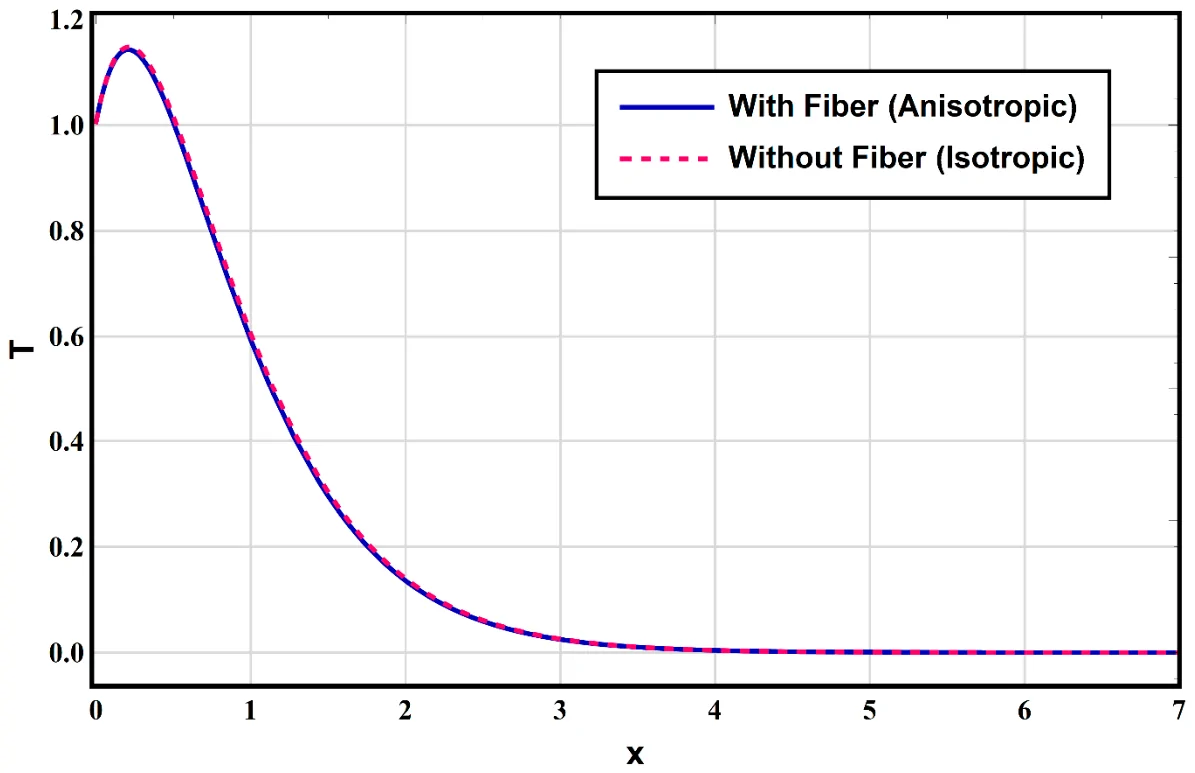
Comparison of the non-dimensional temperature distribution T along the spatial coordinate x between the fiber-reinforced anisotropic and non-reinforced isotropic semiconductor media, illustrating the influence of fiber reinforcement on the thermal response.

**Figure 11:**
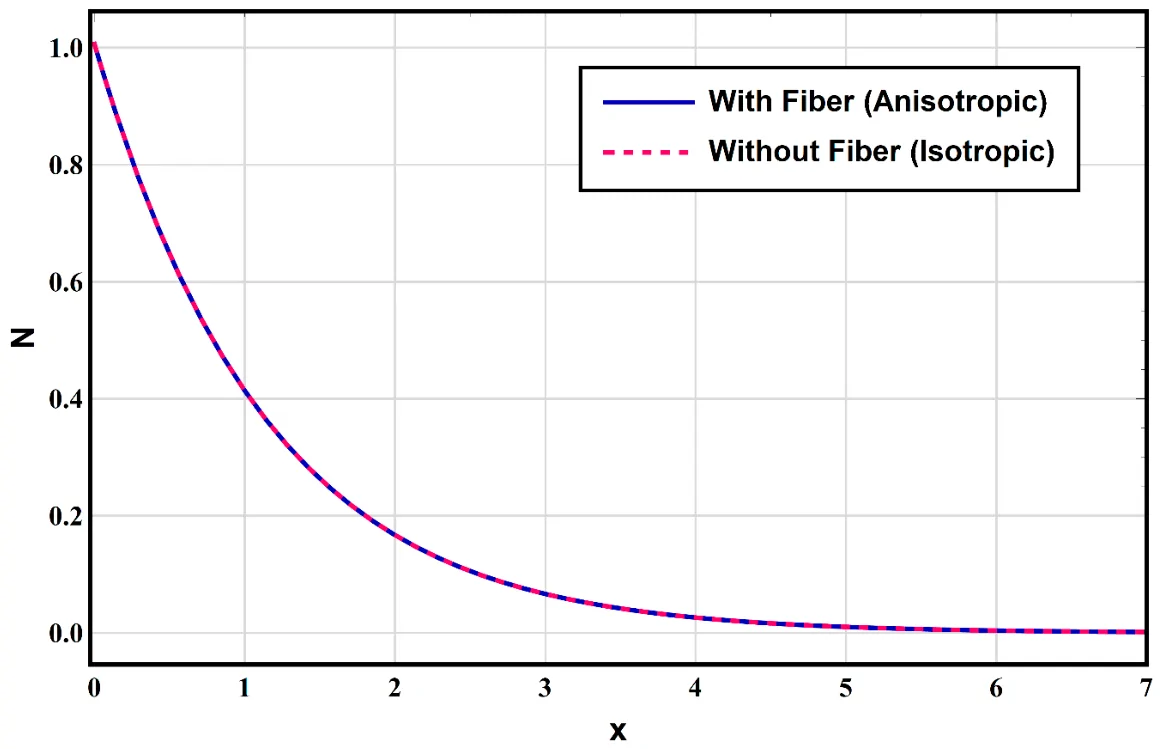
Comparison of the non-dimensional carrier-density distribution N along the spatial coordinate x for the fiber-reinforced anisotropic and non-reinforced isotropic semiconductor media, highlighting the effect of fiber reinforcement on carrier transport.

**Figure 12:**
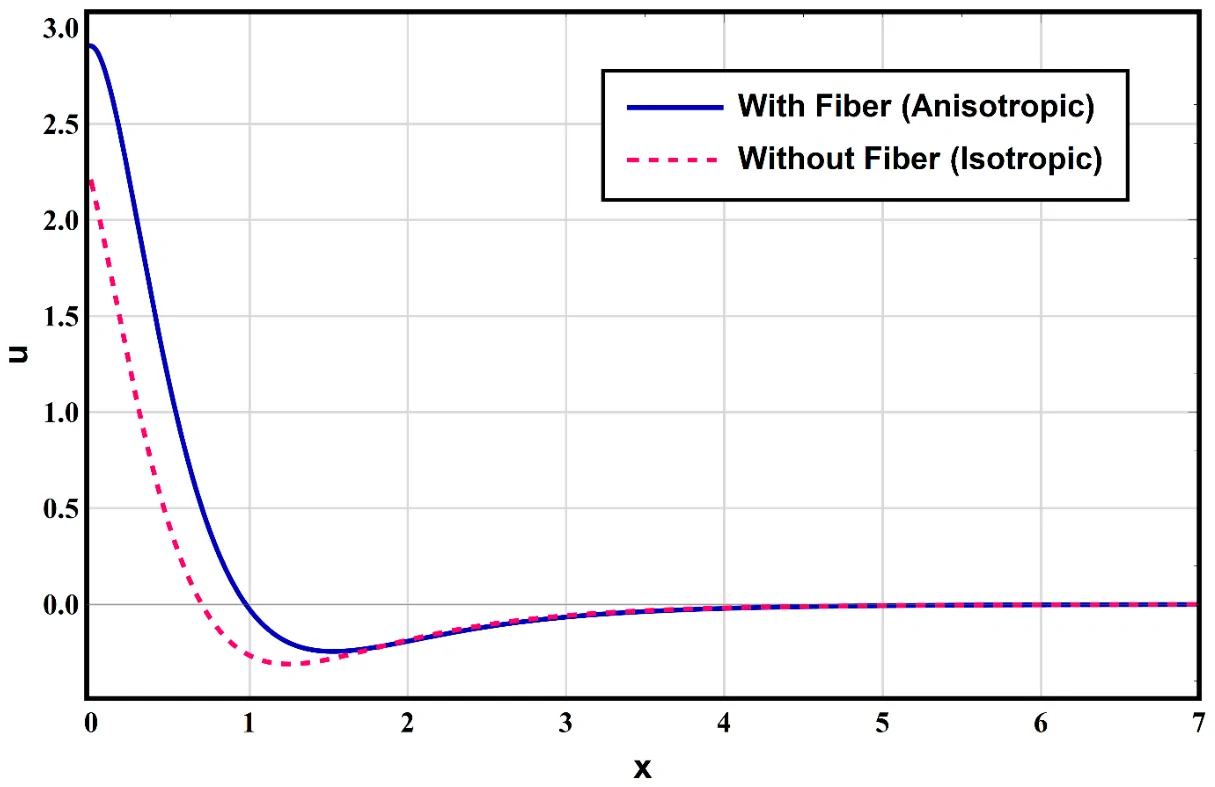
Comparison of the non-dimensional longitudinal displacement u between the fiber-reinforced anisotropic and non-reinforced isotropic semiconductor media, demonstrating the influence of fiber reinforcement on the axial deformation behavior.

**Figure 13:**
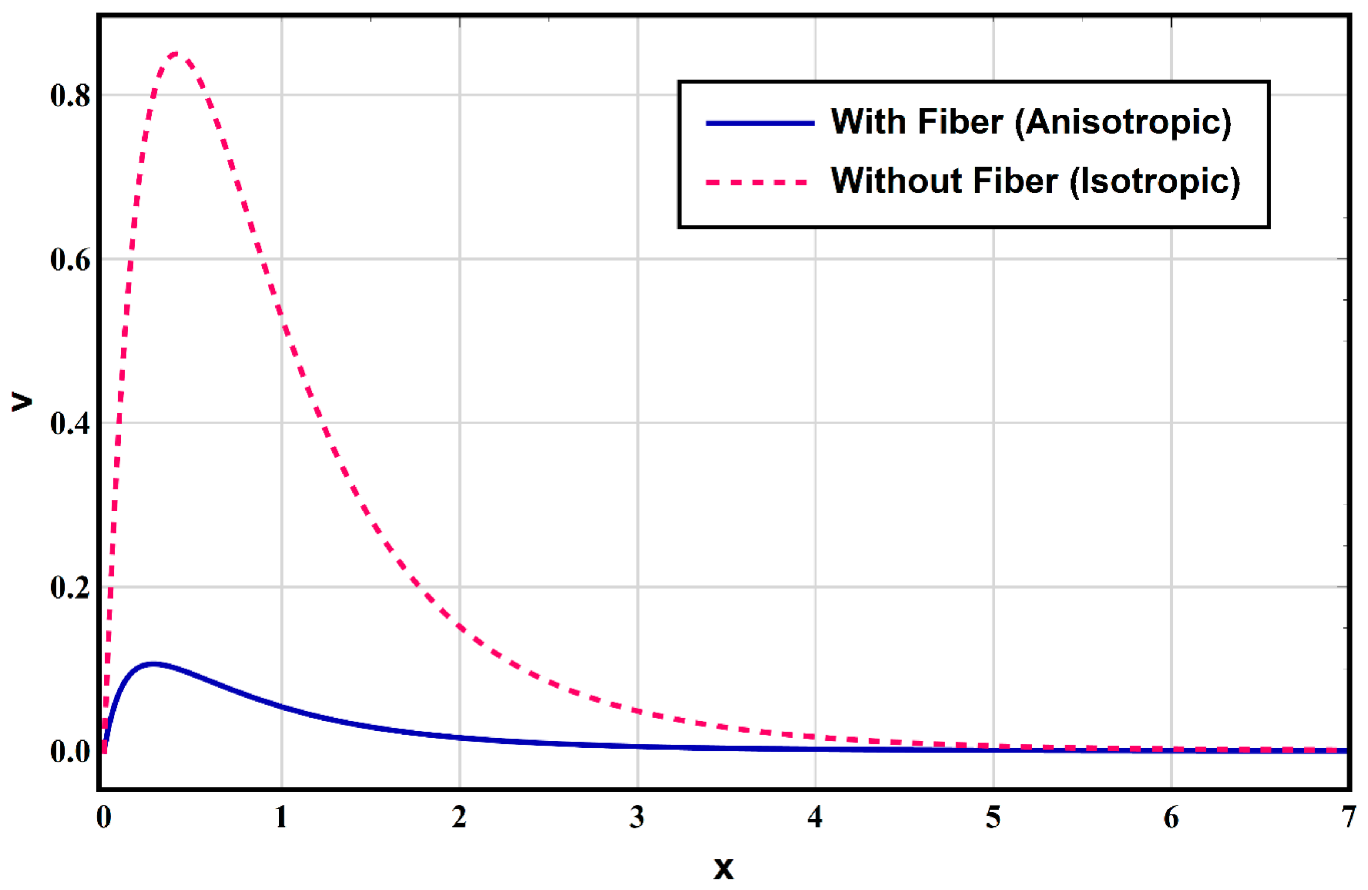
Comparison of the non-dimensional transverse displacement v along the spatial coordinate x for the fiber-reinforced anisotropic and non-reinforced isotropic semiconductor media, showing the effect of fiber reinforcement on the transverse mechanical response.

**Figure 14:**
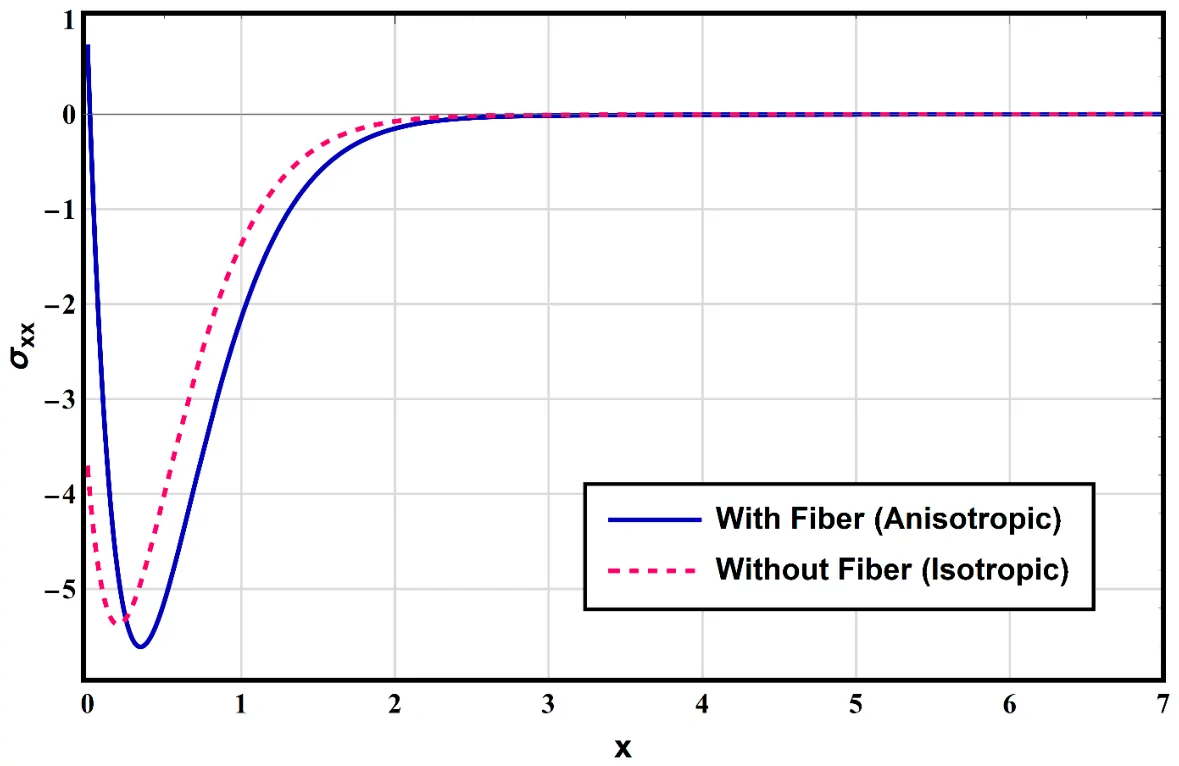
Comparison of the non-dimensional normal stress component $\sigma_{xx}$ between the fiber-reinforced anisotropic and non-reinforced isotropic semiconductor media, illustrating the role of fiber reinforcement in modifying the axial stress distribution.

**Figure 15:**
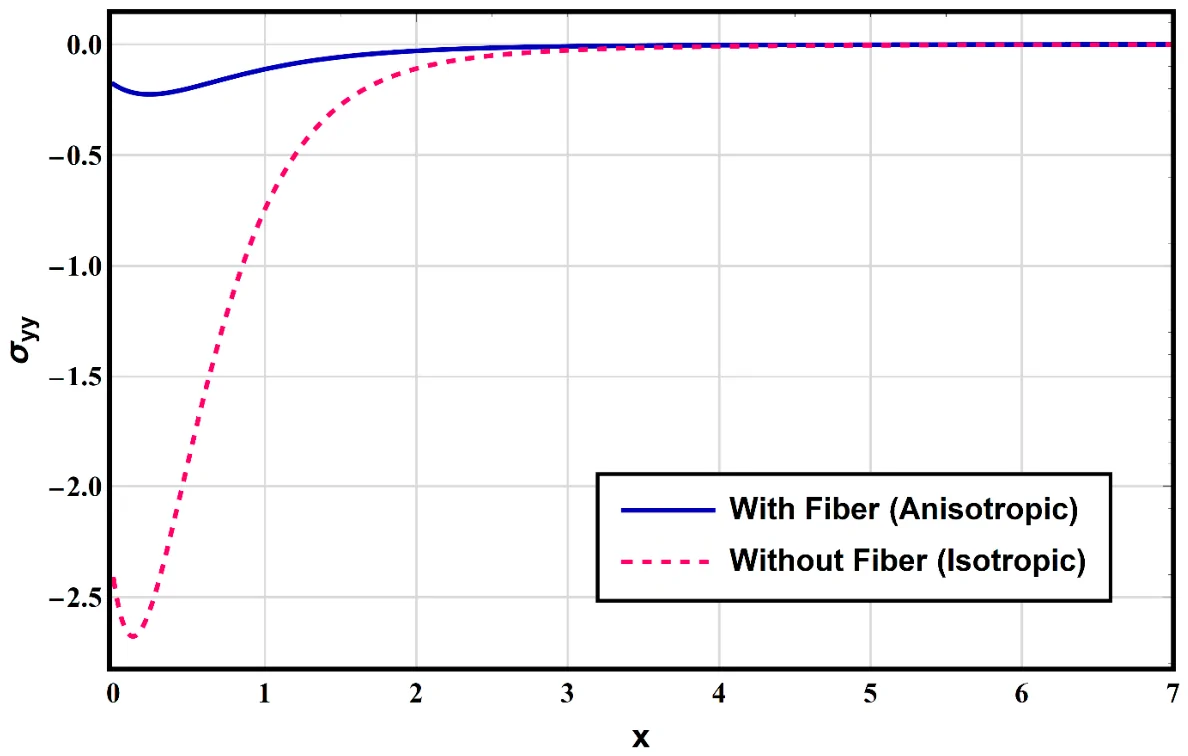
Comparison of the non-dimensional normal stress component $\sigma_{yy}$ along the spatial coordinate x for the fiber-reinforced anisotropic and non-reinforced isotropic semiconductor media, highlighting the influence of fiber reinforcement on the transverse stress response.

**Figure 16:**
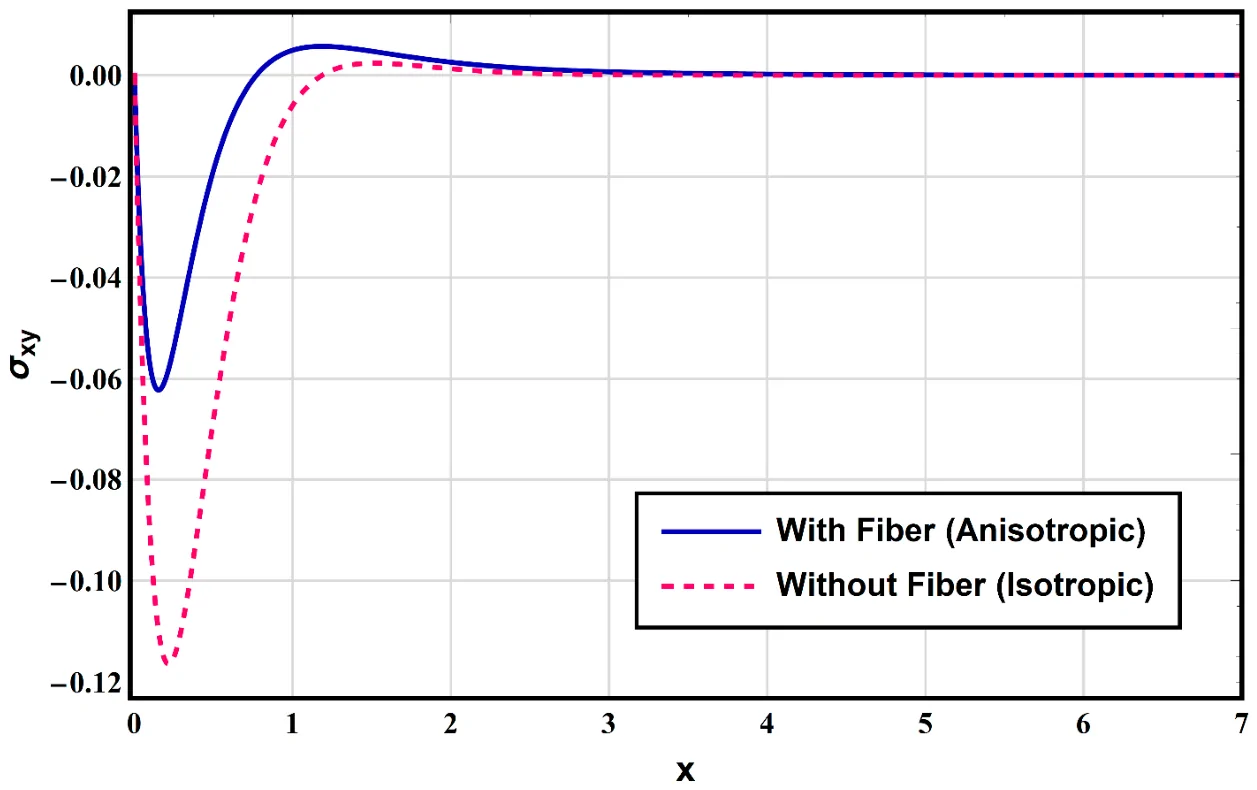
Comparison of the non-dimensional shear stress component $\sigma_{xy}$ between the fiber-reinforced anisotropic and non-reinforced isotropic semiconductor media, demonstrating the effect of fiber reinforcement on the shear stress characteristics of the photo-thermoelastic semiconductor medium.

In this subsection, a comparative study between the fiber-reinforced anisotropic medium and the non-reinforced isotropic medium is presented to investigate the influence of fiber reinforcement on the coupled photo-thermoelastic behavior of the semiconductor material. The obtained graphical distributions are illustrated in Figure 10, Figure 11, Figure 12, Figure 13, Figure 14, Figure 15 and Figure 16 for the temperature T, carrier density N, displacement components u and v, and stress components $\sigma_{xx}$, $\sigma_{yy}$, and $\sigma_{xy}$. The comparison clearly demonstrates that the introduction of reinforcing fibers significantly modifies the mechanical response of the medium, while its influence on the thermal and carrier-density fields remains relatively moderate. This behavior is expected because the reinforcing fibers mainly affect the elastic stiffness and anisotropic mechanical properties of the semiconductor structure. Figure 10 presents the temperature distribution T for both the fiber-reinforced and non-reinforced cases. It is observed that the two curves are very close to each other, indicating that the fiber reinforcement has only a slight influence on the thermal field. The temperature increases near the illuminated surface due to the photothermal excitation and then decays gradually inside the medium because of thermal diffusion. The small difference between the two cases indicates that the heat transfer process is governed mainly by the photothermal and diffusion mechanisms rather than the mechanical anisotropy induced by the fibers. Figure 11 illustrates the carrier density distribution N. Like the temperature field, the carrier density decreases exponentially with increasing distance from the boundary due to carrier recombination and diffusion inside the semiconductor material. The presence of fibers produces only a minor modification in the carrier-density profile. This indicates that the carrier transport process is weakly affected by the anisotropic reinforcement and remains dominated by the semiconductor diffusion mechanisms. The longitudinal displacement component u is shown in Figure 12. In this case, the effect of fiber reinforcement becomes highly significant. The anisotropic fiber-reinforced medium exhibits considerably larger displacement amplitudes near the boundary compared with the isotropic medium. Furthermore, the displacement extrema are shifted, and the attenuation behavior becomes smoother in the reinforced case. This behavior indicates that the fibers modify the stiffness distribution and alter the propagation of elastic waves throughout the medium. The reinforcing fibers enhance the directional mechanical response, leading to stronger coupling between the thermal and elastic fields. Figure 13 represents the transverse displacement component v. A substantial difference between the reinforced and non-reinforced media is clearly observed. The isotropic medium exhibits much larger transverse displacement amplitudes, whereas the fiber-reinforced medium significantly suppresses the transverse motion. This behavior demonstrates that the reinforcing fibers improve the structural stability of the semiconductor medium and reduce excessive transverse deformation. Physically, the fibers act as internal constraints that resist lateral displacement and enhance directional rigidity. The behavior of the normal stress component $\sigma_{xx}$ is illustrated in Figure 14. Both cases exhibit compressive stress behavior near the boundary, followed by gradual decay toward zero at large distances. However, the fiber-reinforced medium produces larger compressive stress magnitudes compared with the isotropic case. This indicates that the reinforcing fibers increase the effective stiffness of the medium and strengthen its resistance against deformation. Consequently, the internal stress concentration becomes more pronounced in the anisotropic structure. Figure 15 shows the variation in the stress component $\sigma_{yy}$. The isotropic medium exhibits significantly larger compressive stress amplitudes than the reinforced anisotropic medium. The reinforcing fibers redistribute the stress field and reduce the magnitude of transverse normal stresses inside the structure. This behavior demonstrates the important role of fiber reinforcement in improving the mechanical stability and controlling stress localization within the semiconductor material. Figure 16 presents the shear stress distribution $\sigma_{xy}$. It is observed that the isotropic medium exhibits larger negative shear stress peaks compared with the reinforced medium. The fiber reinforcement reduces the magnitude of the shear stresses and smooths the stress distribution throughout the domain. This indicates that the anisotropic reinforcement effectively suppresses shear deformation and improves the resistance of the medium against tangential loading effects.

## 13. Physical Interpretation

From the physical viewpoint, the obtained behavior can be explained through the anisotropic mechanical properties introduced by the reinforcing fibers. In the reinforced medium, the fibers provide additional directional stiffness and resistance against deformation. Consequently, the propagation of elastic disturbances becomes highly dependent on the preferred fiber directions, leading to modifications in the displacement and stress distributions compared with the isotropic case. The relatively weak influence of fiber reinforcement on the temperature and carrier-density fields indicates that the thermal and diffusion mechanisms are primarily governed by the semiconductor transport properties rather than the elastic anisotropy. However, the strong influence on the displacement and stress fields demonstrates that the fibers play a dominant role in controlling the mechanical response of the medium. Mathematically, the effect of fiber reinforcement appears through the anisotropic constitutive coefficients introduced into the governing equations. These coefficients modify the coupling terms between the elastic, thermal, and carrier-density fields, leading to changes in the characteristic roots $m_{i}$ and consequently altering the attenuation and propagation behavior of the physical quantities. The reinforced medium therefore exhibits different wave propagation characteristics and stress redistribution mechanisms compared with the isotropic structure. Furthermore, the reduction in transverse displacement and shear stress amplitudes in the reinforced case confirms that the fibers enhance the structural stability and suppress excessive lateral deformation. Such behavior is particularly important in semiconductor and composite-material applications where high mechanical reliability and resistance to thermal deformation are required. From a quantitative perspective, the comparison between the reinforced and isotropic cases demonstrates that fiber reinforcement primarily affects the mechanical response of the medium. The temperature and carrier-density distributions remain almost unchanged, with only negligible differences throughout the domain, confirming that fiber reinforcement has a limited influence on the thermal and diffusion fields. In contrast, the peak longitudinal displacement increases by approximately 30%, whereas the maximum transverse displacement is reduced by nearly 85%, indicating a pronounced suppression of lateral deformation. Similarly, the compressive normal stress $\sigma_{yy}$ is reduced by about 90%, while the peak shear stress $\sigma_{xy}$ decreases by approximately 45%. These quantitative observations confirm that the reinforcing fibers mainly enhance the directional stiffness and mechanical stability of the semiconductor medium, while only marginally influencing the thermal and carrier transport responses.

## 14. Conclusions

In the present work, a coupled photo-thermoelastic model for an anisotropic semiconductor medium reinforced by fibers has been developed within the framework of non-local elasticity. The governing equations describing the thermal, carrier-density, displacement, and stress fields were formulated and transformed into a coupled system of ordinary differential equations using the normal-mode analysis technique. Exact analytical solutions were obtained in terms of eigenvalues and eigenvectors, and the resulting physical fields were evaluated numerically using Mathematica. The numerical results demonstrate that the non-local parameter has a pronounced influence on the mechanical response of the semiconductor medium, particularly on the displacement amplitudes, stress distributions, and attenuation characteristics near the illuminated boundary. In contrast, the temperature and carrier-density distributions exhibit only slight variations with the non-local parameter over the investigated range, indicating that the principal role of nonlocality in the present model is associated with the mechanical response rather than the thermal or carrier-transport fields. These results further highlight the importance of considering small-scale interactions in semiconductor materials, particularly at micro- and nano-scale dimensions, where the predictions of classical local elasticity may become insufficient. A comparative analysis between the fiber-reinforced anisotropic medium and the non-reinforced isotropic medium reveals that fiber reinforcement strongly influences the mechanical response of the structure. Fiber reinforcement reduces transverse deformation and shear-stress amplitudes while improving the mechanical stability and directional stiffness of the medium. The thermal and carrier-density fields, however, remain only slightly affected by the presence of fibers within the investigated configurations, indicating that the principal contribution of fiber reinforcement is associated with the mechanical characteristics of the medium. The obtained results demonstrate that anisotropic fiber-reinforced semiconductor materials possess improved mechanical characteristics compared with conventional isotropic materials. The incorporation of reinforcing fibers modifies the stress redistribution, suppresses excessive deformation, and enhances the structural response under photothermal loading conditions. These characteristics make fiber-reinforced anisotropic materials potentially useful for advanced engineering and technological applications involving semiconductor devices, optoelectronic systems, laser-heated materials, nano-scale thermal sensors, microelectronic structures, smart composite materials, and nano-electromechanical systems (NEMS). From a practical design perspective, the present findings suggest that fiber orientation and reinforcement ratio can be tailored to enhance the mechanical stability of optoelectronic composite components without significantly altering their thermal or carrier-transport performance, offering a useful design guideline for engineers developing semiconductor-based photothermal devices where mechanical robustness under optical loading is a key requirement. The present analytical framework can also be extended in several promising directions. Future work may consider extending the model to finite-thickness layered semiconductor structures, more representative of real device geometries than the semi-infinite configuration considered here; incorporating temperature-dependent material coefficients to capture the nonlinear thermophysical behavior of semiconductors under strong photothermal excitation; and combining the present analytical approach with finite-element numerical methods to accommodate complex device geometries and boundary configurations beyond the reach of closed-form analytical solutions. Such extensions would provide further insight into the coupled thermal, carrier, and mechanical behavior of advanced semiconductor structures. Overall, the present investigation demonstrates that the combined consideration of non-local elasticity and fiber reinforcement provides a more comprehensive description of coupled photo-thermoelastic behavior in anisotropic semiconductor materials, particularly for applications involving micro- and nano-scale structures.

## Figures and Tables

**Table 1:** Quantitative comparison of the temperature field T in the local limiting case (ξ=0) between the present study and Ref [43] at t=0.9.

x	T Present Study (ξ=0)	T Ref. [43]	Relative Difference (%)
0.00	1.006236	1.006236	0.000
0.01	1.020681	1.020370	0.030
0.02	1.034136	1.033534	0.058
0.03	1.046636	1.045762	0.084
0.04	1.058213	1.057086	0.107

**Table 2:** Material properties and corresponding constants of silicon (Si) used in the present analysis, given in SI units.

Symbol	Si	Unit
λ	$3.64\times 10^{10}$	$N/m^{2}$
$\mu_{T}$	$5.46\times 10^{10}$	$N/m^{2}$
$\mu_{L}$	$3.20\times 10^{10}$	$N/m^{2}$
ρ	2330	$kg/m^{3}$
$C_{E}$	695	J/(kg⋅K)
$k_{11}$	$0.0921\times 10^{3}$	W/(m⋅K)
$k_{22}$	$0.0963\times 10^{3}$	W/(m⋅K)
$D_{E}$	$2.5\times 10^{-3}$	$m^{2}/s$
τ	$5\times 10^{-5}$	s
$T_{0}$	300	K
$E_{g}$	$1.11\times 10^{-19}$	J
$\alpha_{11}$	$3.1\times 10^{-6}$	$K^{-1}$
$\alpha_{22}$	$3.5\times 10^{-6}$	$K^{-1}$
$\kappa =\left(\partial N_{0}/\partial T\right)(1/\tau )$	$2.16\times 10^{21}$	$m^{-3}\cdot K^{-1}\cdot s^{-1}$
α	$-1.28\times 10^{10}$	$N/m^{2}$
β	$220.90\times 10^{10}$	$N/m^{2}$
$\xi_{11}$	$-7\times 10^{-31}$	$m^{3}$
$\xi_{22}$	$-9\times 10^{-31}$	$m^{3}$
ω	2.95+i	$s^{-1}$
a	1	$m^{-1}$
y	0.6	m
$\theta_{0}$	1	dimensionless
$N_{0}$	1	dimensionless

## Data Availability

The data that support the findings of this study are available from the corresponding author upon reasonable request.
